# Supplementary material for: Probabilistic seasonal dengue forecasting in Vietnam: A modelling study using superensembles
Source: PLoS Med. 2021 Mar 4;18(3):e1003542. doi: 10.1371/journal.pmed.1003542 (PMC7971894; doi:10.1371/journal.pmed.1003542)
Supplement: S1 Text — (DOCX) [file pmed.1003542.s001.docx]

Probabilistic seasonal dengue forecasting in Vietnam: A modelling study using superensembles

Felipe J Colón-González, Leonardo Soares Bastos, Barbara Hofmann, Alison Hopkin, Quillon Harpham, Tom Crocker, Rosanna Amato, Iacopo Ferrario, Francesca Moschini, Samuel James, Sajni Malde, Eleanor Ainscoe, Vu Sinh Nam, Dang Quang Tan, Nguyen Duc Khoa, Mark Harrison, Gina Tsarouchi, Darren Lumbroso, Oliver Brady, Rachel Lowe

**S1 Text. Prospective analytical plan**

The analysis undertaken in this manuscript was described in a UK Space Agency grant proposal for the International Partnership Programme in September 2017.

**Project Title: An integrated dengue early warning system driven by Earth Observations in Vietnam**

**Background**

Dengue fever occurs in over 100 countries (Kraemer et al., 2015) where it is disproportionately linked to poverty and inequality. Climatic factors such as temperature, precipitation, and humidity modulate many aspects of the ecology of the disease such as the reproduction rate of the vector and the transmission rate of the virus. Thus, the climatic conditions of a region or time period may be indicative of dengue risk in the near or far future (e.g. Colón-González, et al., 2013, 2018). Research has highlighted the potential usefulness of seasonal-climate-driven epidemiological surveillance for decision-making and planning (Lowe et al., 2013, 2014, 2017). Yet, the integration of climate-driven early warning systems into existing public health procedures has been difficult.

Currently, Vietnam lacks an early warning system to forecast dengue risks. In recent years, the health systems in Vietnam have struggled to maintain adequate control of the dengue vectors *Aedes aegypti* and *Aedes albopictus*. The outputs generated in this project will provide a tool to give users advance warning of dengue risk several months in advance. This information will greatly support public health decision-making, particularly with regard to the administration and allocation of scarce resources to areas where they are most needed. Earth observation data will be combined with health and water availability information to generate an integrated dengue forecasting system (Lowe et al., 2013, 2014, 2017; Machault et al., 2014; Moreno-Madriñán, et al., 2014). The integrated system will link Earth observations with seasonal climate forecasting and a land-surface model to understand and predict the impacts of important determinants of dengue risk and the likelihood of future dengue outbreaks.

**Aims**

- Develop an early warning system to improve the prevention of dengue outbreaks and increase response capacity in Vietnam;
- Create a better understanding of the relationships between dengue risk and a set of environmental factors including the hydrological-climate system; and
- Provide a range of scenarios of dengue risk to guide decision-making and planning.

**Methods**

The early warning system will be based on a suite of statistical forecasting models of dengue risk based on a spatio-temporal Bayesian hierarchical mixed modelling approach. The system will incorporate environmental covariates important for dengue transmission to forecasts dengue incidence up to six months ahead. These variables will be related to the ecology of the main vector *Aedes aegypti* and will be included to strengthen the model and enhance its ability to forecast dengue outbreaks. The fully integrated dengue early warning system will include:

- Seasonal forecasts of meteorological variables (e.g. temperature, rainfall, relative humidity), which are important factors in the ecology of the disease;
- Socio-economic indicators related to urbanisation;
- Historical reports of dengue cases.

Probabilistic spatio-temporal models will be used to fit linear relationships between dengue incidence for the period 1993-2010 and each of the covariates for all 63 provinces of Vietnam. The models will provide predictions of monthly dengue incidence and its corresponding uncertainty level and the probability of exceeding a pre-defined outbreak threshold.

**References**

Colón-González FJ, et al. (2018) Limiting global-mean temperature increase to 1.5–2 °C could reduce the incidence and spatial spread of dengue fever in Latin America. PNAS 115(24): 6243-6248.

Colón-González FJ, et al. (2013) The Effects of Weather and Climate Change on Dengue. PLoS Negl Trop Dis 7(11): e2503.

Goetz, S.J. et al. (2000). Advances in satellite remote sensing of environmental variables for epidemiological applications. Adv. Parasitol., 47, 289–307

Kraemer M.U., et al. (2015). The global distribution of the arbovirus vectors *Aedes aegypti* *and Ae. albopictus*. Elife, 4:e08347.

Lowe R, et al. (2017). Climate services for health: predicting the evolution of the 2016 dengue season in Machala, Ecuador. Lancet Planetary Health.

Lowe R, et al. (2014). Dengue outlook for the World Cup in Brazil: an early warning model framework driven by real-time seasonal climate forecasts. Lancet Infectious Diseases, 14(7)

Lowe R, et al. (2013). The development of an early warning system for climate-sensitive disease risk with a focus on dengue epidemics in Southeast Brazil. Statistics in Medicine, 32(5): 864–883

Machault, V. et al. (2014). Mapping entomological dengue risk levels in Martinique using high-resolution remote-sensing environmental data. ISPRS Int. J. Geo-Inf. 3, 1352–1371

Moreno-Madriñán, M. et al. (2014). Correlating remote sensing data with the abundance of pupae of the dengue virus mosquito vector, *Aedes aegypti*, in central Mexico. ISPRS Int. J. Geo-Inf. 3, 732–749
