# Supplementary figures and images for: Probabilistic seasonal dengue forecasting in Vietnam: A modelling study using superensembles
Source: PLoS Med. 2021 Mar 4;18(3):e1003542. doi: 10.1371/journal.pmed.1003542 (PMC7971894; doi:10.1371/journal.pmed.1003542)

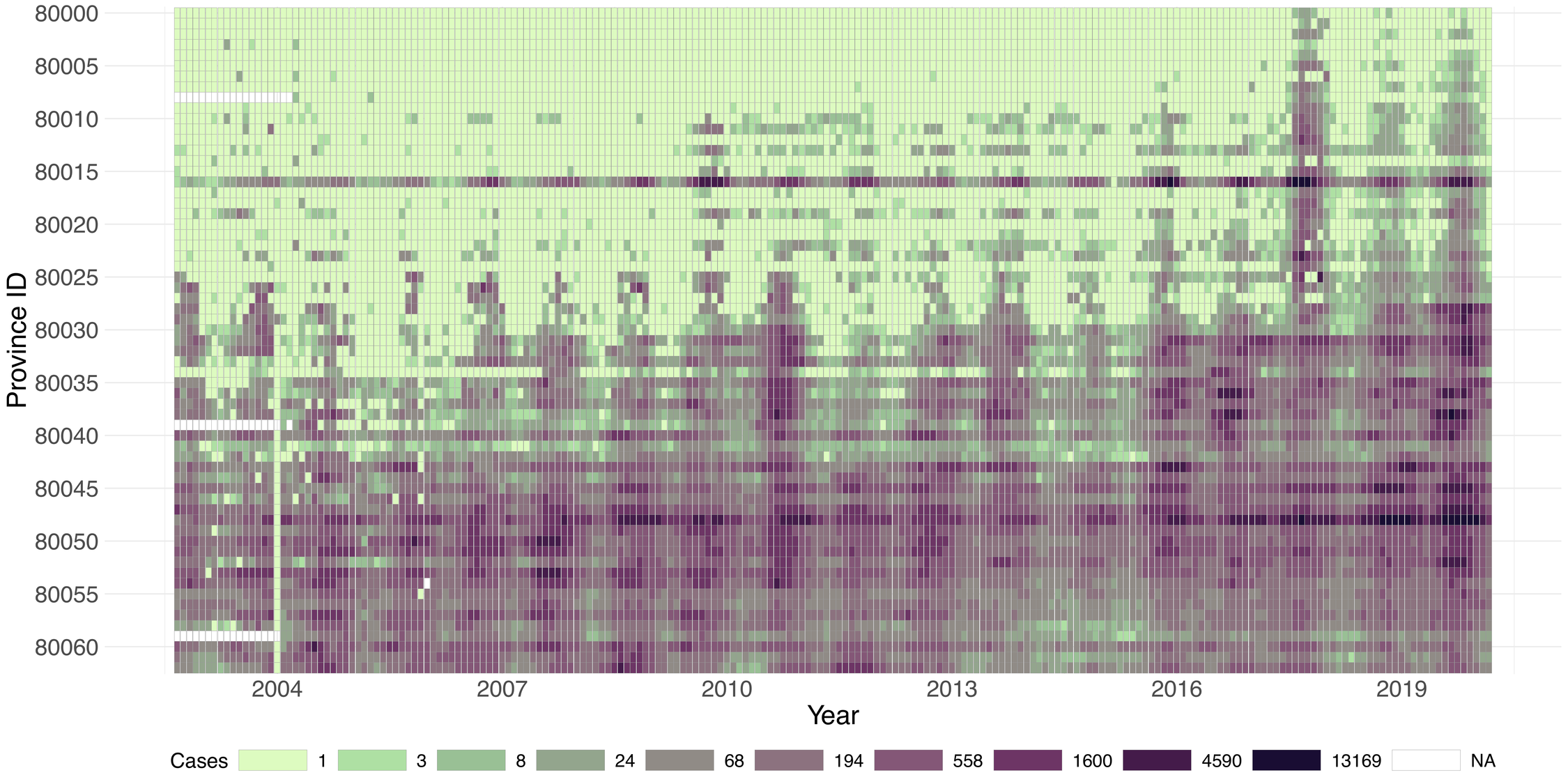

Supplement: S1 Fig — Time series of monthly dengue cases from the 63 provinces in Vietnam (August 2002 to March 2020). Provinces are ordered from north (top) to south (bottom) according to the latitude coordinates of their centroid. White boxes indicate missing data. (TIF) [file pmed.1003542.s002.tif]

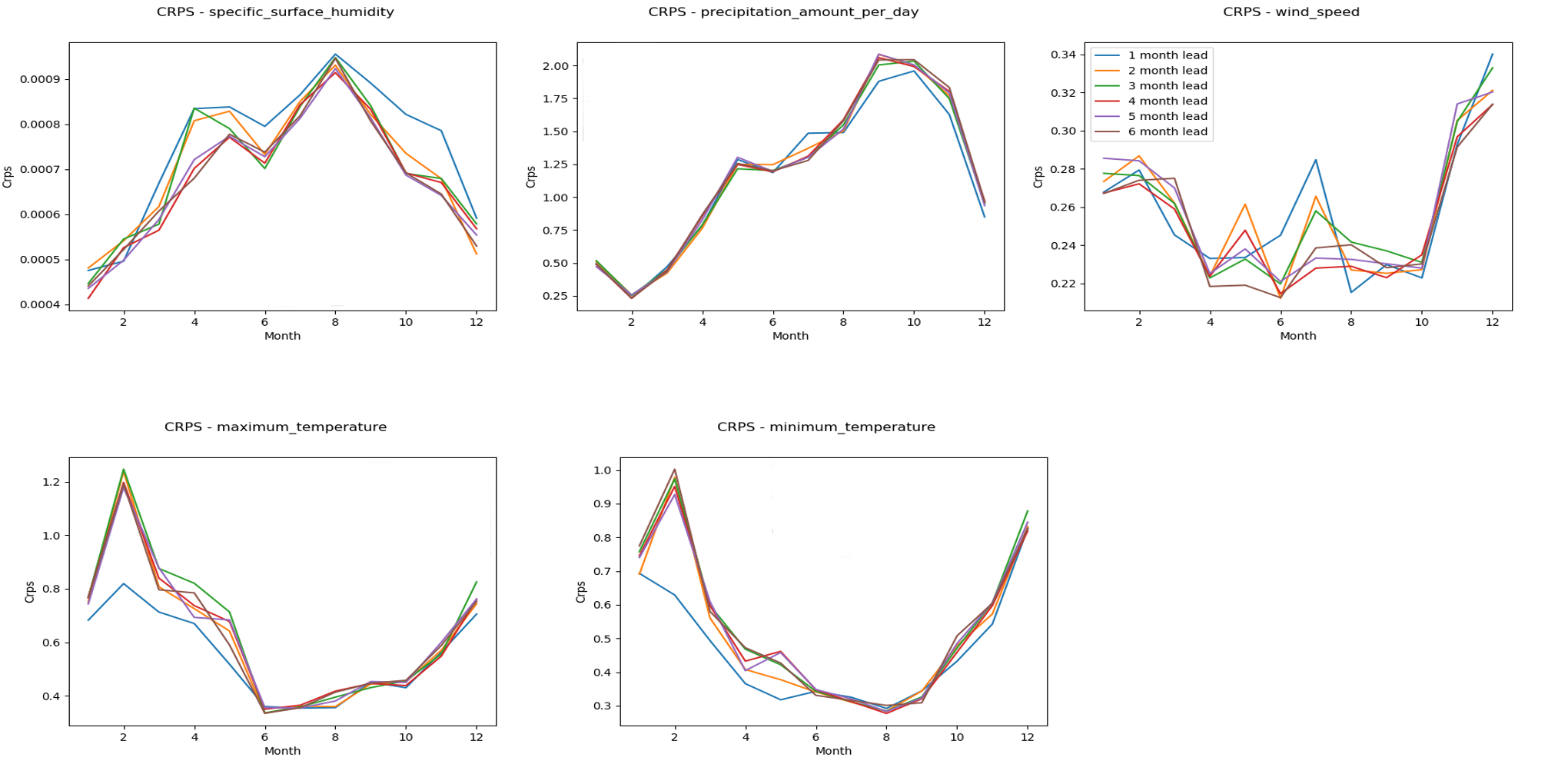

Supplement: S2 Fig — The x axis indicates the month of the year. The y axis indicates value of the CRPS for each variable. The lines indicate the lead time for the forecast. CRPS, continuous rank probability score. (TIF) [file pmed.1003542.s003.tif]

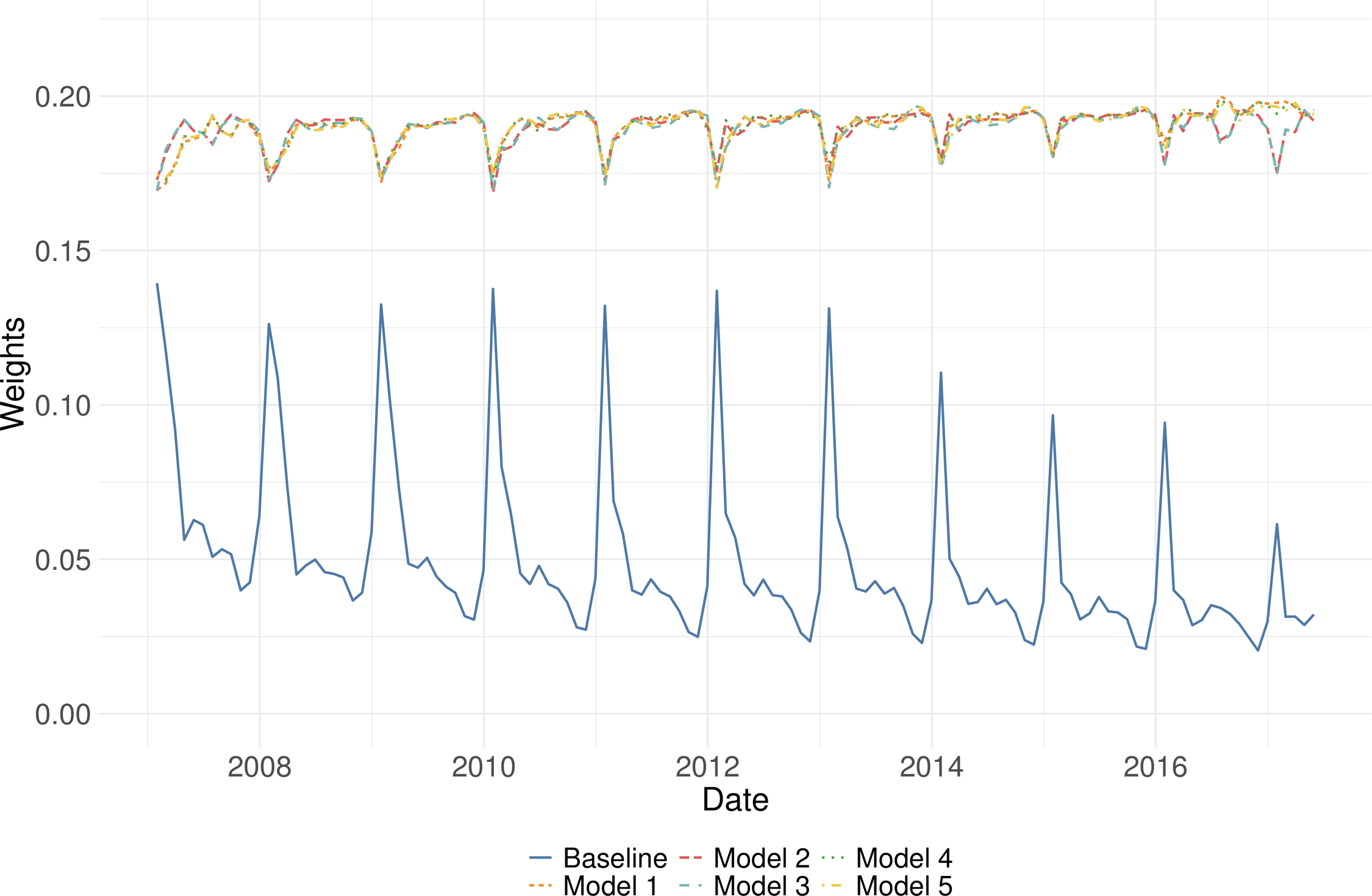

Supplement: S3 Fig — Seasonal variation of the optimal weights assigned to each of the models included in the superensemble over the period January 2007 to December 2016. (TIF) [file pmed.1003542.s004.tif]

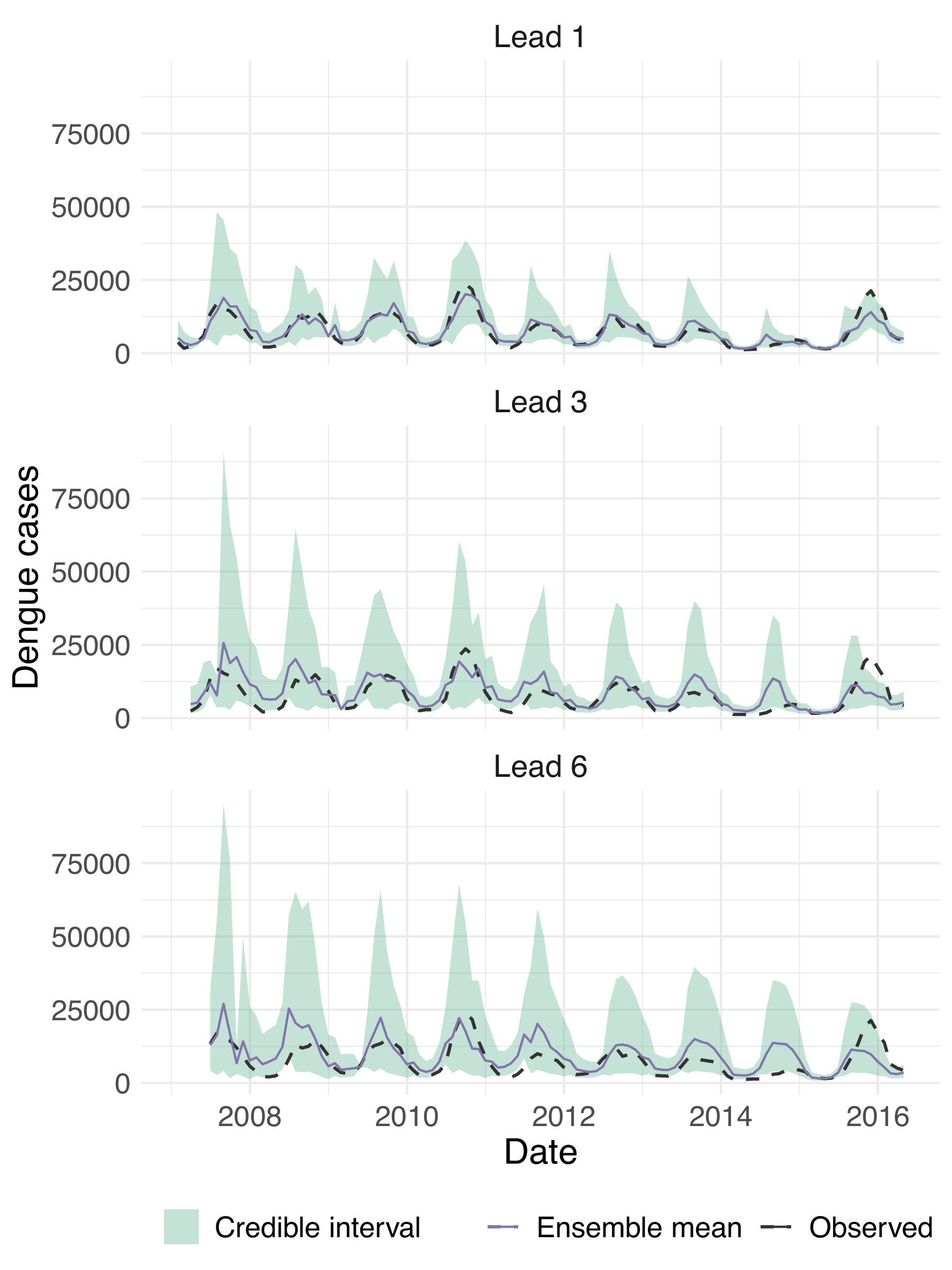

Supplement: S4 Fig — Observed (dashed lines) and predicted (solid lines) dengue cases across Vietnam aggregated at the national level. Shaded areas represent the 95% credible interval. Predictions are shown for the forecast horizons of 1 (top), 3 (middle), and 6 (bottom) months ahead. Data correspond to the period January 2007 to December 2016. (TIF) [file pmed.1003542.s005.tif]

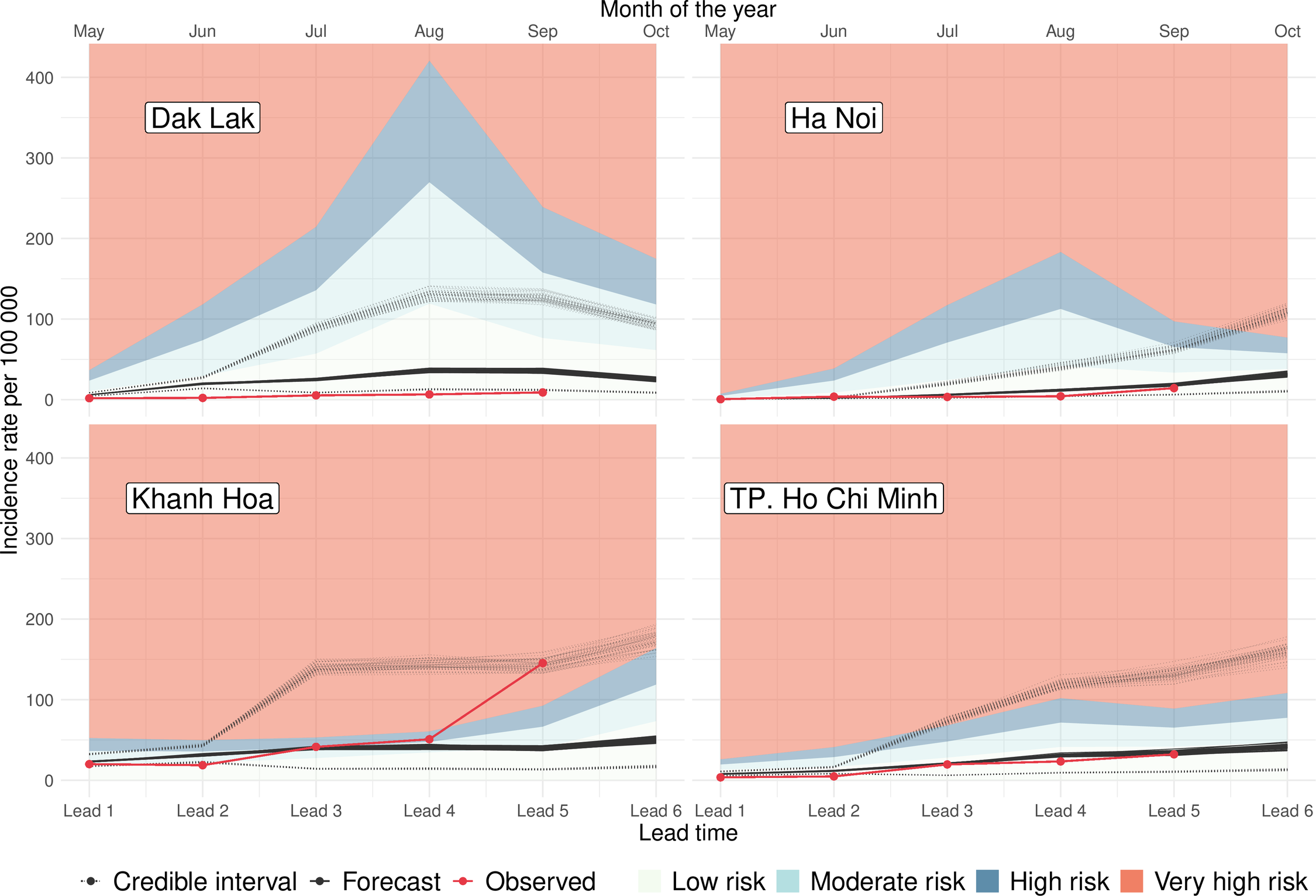

Supplement: S5 Fig — Predicted dengue cases for the period May to October 2020 for 4 pilot Vietnamese provinces using a model superensemble. The forecast was issued on May 10, 2020. The x axis (top) indicates the month of the predictions. The x axis (bottom) indicates the time lead of the predictions. The y axis indicates the predicted incidence rate. Black solid lines indicate the posterior mean estimate for each of 42 forecast ensemble members. Red lines indicate the observed dengue incidence rate which was not known by the model at the time of the computation and are included here as a reference. The black dashed lines indicate the upper and lower bounds of the 95% credible intervals for the 42 ensemble members. The upper bound of the shaded areas indicates the month- and province-specific percentiles based on dengue data for previous 5 years. (TIF) [file pmed.1003542.s006.tif]

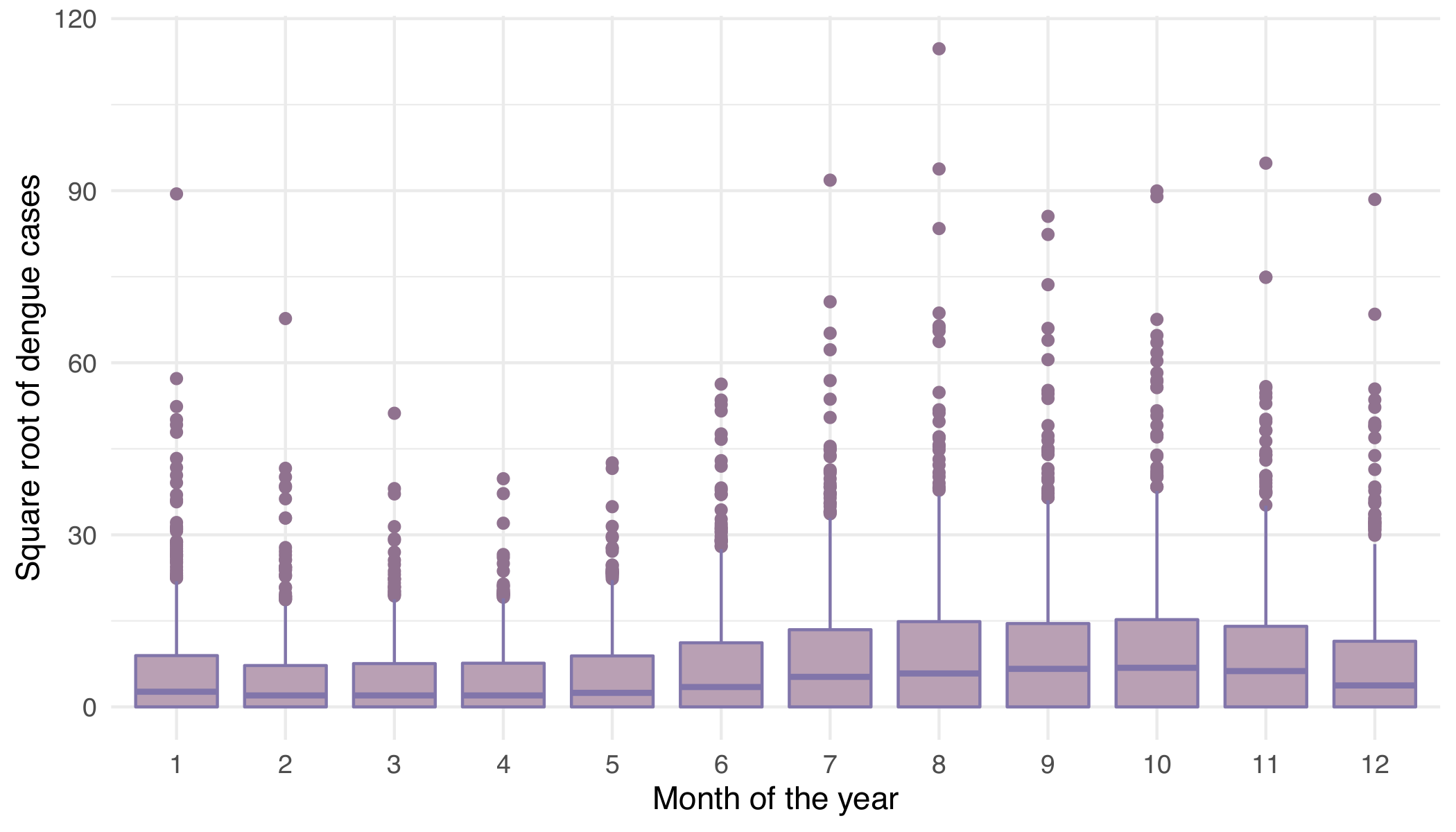

Supplement: S6 Fig — The x axis indicates the month of the year. The y axis indicates increases in the number of dengue cases (square root transformed). The upper and lower limits of each box represent the interquartile range of the distribution of dengue cases for each month. The middle solid line indicates the median value. The upper and lower whiskers indicate the maximum and minimum values of the dengue case distribution (excluding outliers which are indicated with dark purple circles). Outliers are values beyond 1.5 times the interquartile range. (TIF) [file pmed.1003542.s007.tif]
