# Supplementary material for: Probabilistic seasonal dengue forecasting in Vietnam: A modelling study using superensembles
Source: PLoS Med. 2021 Mar 4;18(3):e1003542. doi: 10.1371/journal.pmed.1003542 (PMC7971894; doi:10.1371/journal.pmed.1003542)
Supplement: Attachment — Submitted filename: Response_to_reviewers.docx [file pmed.1003542.s008.docx]

**Response to editor and reviewer comments**

**Manuscript reference number:** PMEDICINE-D-20-02989R1

**Title:** Probabilistic seasonal dengue forecasting in Vietnam: A modelling study using superensembles

We wish to thank the editors and all reviewers for their valuable comments on our manuscript which have led to several improvements to the analysis and manuscript. All changes are described below and can be summarised as follows:

- We incorporated the calculation of the bias and sharpness of each of the competing models included in the superensemble to decompose the overall prediction error.
- We modified the specification of the climate naïve baseline model to a model that captures the spatial dependency and seasonality for each province. This baseline model predicts the same seasonal average of dengue incidence each month.
- The specification of the model superensemble was modified from using Bayesian moving averaging to model stacking based on the continuous rank probability score.
- The specification of the weighting of each model included in the superensemble changed from using weights based on maximum likelihood to an approach based on the variance of errors.
- We modified the colour palette and the methodology used for communicating and visualising the outputs from the probabilistic superensemble.

**Requests from the editors:**

- **Please revise your title according to PLOS Medicine's style - this should summarise the study question but also state (preferably after colon in the subtitle) the study design (e.g. "A randomized controlled trial," "A retrospective study," "A modelling study," etc.).**

Thank you for this important request. We have amended the title of the manuscript as follows (Page 1):

*“Probabilistic seasonal dengue forecasting in Vietnam: A modelling study using superensembles”*

- **In the last sentence of the Abstract Methods and Findings section, please add a brief summary of any key limitation(s) of the study's methodology.**

We have included the following at the end of the Abstract Methods and Findings section (Abstract, page 1):

*“Despite the demonstrated value of our forecasting system, the approach is limited by the consistency of the dengue case data, as well as the lack of publicly available, continuous, and long-term data sets on mosquito control efforts and serotype-specific case data..”*

- **At this stage, we ask that you include a short, non-technical Author Summary of your research to make findings accessible to a wide audience that includes both scientists and non-scientists. The Author Summary should immediately follow the Abstract in your revised manuscript. This text is subject to editorial change and should be distinct from the scientific abstract. Please see our author guidelines for more information:**

[**https://journals.plos.org/plosmedicine/s/revising-your-manuscript#loc-author-summary**](https://journals.plos.org/plosmedicine/s/revising-your-manuscript#loc-author-summary)

We have added the following Author Summary to the manuscript (pages 2-3):

***Why was this study done?***

- *A climate-driven dengue early warning system would allow public health decision-makers to design, implement, and target timely interventions to the most at-risk places.*
- *Dengue is sensitive to changes in temperature, rainfall, humidity and wind speed. Therefore, a dengue prediction model driven by seasonal climate forecasts offers a valuable tool for predicting dengue risk in advance.*

***What did the researchers do and find?***

- *We developed a superensemble of probabilistic models to predict dengue incidence across Vietnam up to six months ahead. The predictive ability of the superensemble was assessed using multiple verification metrics, and compared to a baseline model at multiple lead times, seasons, and locations.*
- *We found that the model superensemble generated more accurate predictions than the baseline model one to three months ahead but not four to six months ahead.*
- *The model superensemble, however, was better able to predict outbreaks than the baseline model across all lead-times.*
- *The predictive ability of the model varied with geographic location, forecast horizon, and time of the year, and performed best in the peak season in areas experiencing a high level of transmission.*

***What do these findings mean?***

- *Whilst outbreaks of infectious diseases are difficult to predict, particularly several months ahead, model superensembles, which combine multiple climatic drivers with dengue surveillance data at the time of forecast issue date, provide a useful decision-support tool.*
- *Early warning systems driven by seasonal climate forecasts could shift dengue control towards a more preventative approach, guiding bespoke and targeted public health interventions, and a more efficient allocation of scarce resources.*

**Please clarify whether the analytical approach detailed here was laid out in a prospective protocol or analysis plan? Please state this (either way) early in the Methods section.**

a) **If a prospective analysis plan (from your funding proposal, IRB or other ethics committee submission, study protocol, or other planning document written before analyzing the data) was used in designing the study, please include the relevant prospectively written document with your revised manuscript as a Supporting Information file to be published alongside your study, and cite it in the Methods section. A legend for this file should be included at the end of your manuscript.**

We have incorporated the following at the beginning of the **Methods** section (pages 5-6, lines 140-153):

- *“The analysis plan was described in a UK Space Agency grant proposal for the International Partnership Programme in September 2017. The aims, objectives, and proposed methodology were developed prior to data analysis and are described in S1 Text. Changes to the analytical plan in response to suggestions from reviewers took place between August and October 2020. These changes include: (1) calculating the bias and sharpness of each of the competing models included in the superensemble to decompose the overall prediction error; (2) formulating a climate naïve baseline model that captures the spatial dependency and seasonality for each province and estimates the same seasonal average of dengue incidence each month; (3) modifying the model superensemble specification from Bayesian moving averaging to model stacking based on the continuous rank probability score; (4) using weights based on the variance of errors rather than maximum likelihood; (5) modifying the colour palette and the methodology used for communicating and visualising the outputs from the probabilistic superensemble.”*

b) **If no such document exists, please make sure that the Methods section transparently describes when analyses were planned, and when/why any data-driven changes to analyses took place.**

We have incorporated **S1Text** detailing the prospective analytical plan.

c) **In either case, changes in the analysis-- including those made in response to peer review comments-- should be identified as such in the Methods section of the paper, with rationale.**

Changes to the analytical plan have been identified in the manuscript as post hoc analyses.

*“In response to peer review comments, we also evaluated the bias of the forecasts with the lowest CRPS to investigate whether models systematically over- or under-predicted dengue counts [74].”* (page 9, lines 299-301)

*“We developed a historical baseline model, which forecasts the same seasonal average of dengue incidence every month at each model run following [17,33,77] to compare all models to a common reference.”* (page 10, lines 390-392	)

*“Given a number of competing models, we generated a model superensemble [37] stacking models according to their CRPS. Stacking involves averaging point estimates from multiple models using weighted averages. We found model stacking outperformed Bayesian moving average. Therefore, this technique was adopted for the final version of the system.”* (page 11, lines 362-366)

**Comments from the reviewers:**

**Reviewer 1:**

- **Dengue is a climate-sensitive disease, and many efforts have been attempted to predict future outbreak risk using environmental conditions and past patterns of dengue. However, it is relatively scarce to combine forecasts from multiple competing models into a superensemble to make more accurate predictions. This study aimed to develop a superensemble of probabilistic hierarchical dengue models to predict dengue risk up to six months ahead at the province level across Vietnam. It is an interesting and well-presented manuscript but several issues should be addressed to improve its clarity.**

We thank the reviewer for this positive evaluation of our manuscript.

- **How were suspected and confirmed dengue cases diagnosed and reported? More information on each category is clearly needed. The quality and consistency of the dengue data was likely to vary with area and time, and it remains largely unknown how this problem might affect the construction of the model.**

We have incorporated the following text in the **Dengue Surveillance Data** **sub-section** to clarify how suspected and confirmed cases are reported in the country (page 6, lines 160-171).

*“According to the national guidelines on dengue surveillance, suspected cases are defined as people living in or coming from endemic areas or from areas that have had dengue outbreak foci over the previous 14 days with manifestation of sudden high fever lasting for two to seven days, and with at least two of the following signs: hemorrhagic manifestation (positive tourniquet test, hypodermic petechia or ecchymosis, stump hemorrhage or nasal hemorrhage), headache, anorexia, nausea, vomiting, skin flush, rash, muscular, joint and orbital pains, writhe, unconsciousness, pain in liver area or pain when pressing on the liver area, hepatomegaly, thrombocytopenia, or increased hematocrit. Confirmed cases correspond to those confirmed by laboratory tests using Mac - Elisa, PCR, and NS1, or virus isolation. Over the period 2002-2019, there have been no changes in the classification of suspected and confirmed dengue cases in Vietnam.”*

- **Data on both disease and environmental conditions (e.g. meteorological, demographic and land-cover variables) had different spatial resolutions and it is unclear how these datasets were matched.**

We thank the reviewer for highlighting this issue. We have now incorporated an explanation as to how different data were reconciled (page 7, lines 196-217).

*“Earth observation data were obtained as a gridded dataset in netcdf format. Socio-demographic data were averaged at the province level using a user-defined function in Python 3.6 [63]. When a polygon covered only part of a grid cell, averages were calculated for the approximate fraction of the cell covered by the polygon (rounded 1/100). The administrative boundaries of each province were defined using a shapefile provided by the Vietnamese General Department of Preventive Medicine, Hanoi.*

*Climate data were aggregated at the province level using population weighted averages. Population data from the WorldPop project [64] at a 100 m^2^ spatial resolution was used to calculate annual gridded weights for each province using Python 3.6 [63]. At the time of processing, WorldPop data were only available for the years, 2009, 2010, 2015, 2020. Intervening years were calculated using linear interpolation. Province-specific population-weighted averages were calculated as follows:*

$$\hat{x}= \frac{\sum_{i=1}^{n} w_{i}x_{i}}{\sum_{i=1}^{n} w_{i}},$$

*where n is the total number of pixels i falling within a given province polygon, x is the value of each climate predictor at pixel i, and w indicates the pixel-specific weights calculated as:*

$$w_{i}= \frac{p_{i}}{N},$$

*where p_i_ is the pixel-specific population count, and N is the total population count falling into a given province.”*

- **Is there any change in the administrative boundary across 63 provinces in Vietnam during the period 2002-2019?**

Over the study period the following changes happened in the administrative boundaries:

1. Nov 2003: Lai Chau province was divided into 2 provinces, Dien Bien and Lai Chau.
2. Nov 2003: Dak Lak province was divided into 2 provinces, Dak Nong and Dak Lak
3. Nov 2003: Can Tho province was divided into 2 provinces, Can Tho city and Hau Giang province
4. In 2008: Ha Tay province merged with Ha Noi city to become Ha Noi

To ensure consistency across time and space, we froze the administrative boundaries of each province to their 2008 level. Dengue data were allocated to each of the newly created provinces using district-level records. This is now explained in the manuscript as follows (page 7, lines 218-223):

*“There were multiple changes in the administrative boundaries of the provinces over the study period. The last change took place in 2008. Consequently, we froze the administrative boundaries of the country at their 2008 level. Dengue data were allocated to each of the 2008-level administrative boundaries using district-level records. The allocation of dengue cases to each province was conducted by the General Department of Preventive Medicine, Hanoi.”*

- **How were population weighted monthly averages calculated for each climate variable?**

We have added the following text to the **Historical Earth Observation data sub-section** (page 7, lines 212-215):

*“Province-specific population-weighted averages for each province were calculated as follows:*

$$\hat{x}= \frac{\sum_{i=1}^{n} w_{i}x_{i}}{\sum_{i=1}^{n} w_{i}},$$

*where n is the total number of pixels i falling within a given province polygon, x is the value of each climate predictor at pixel i, and w indicates the pixel-specific weights calculated as:*

$$w_{i}= \frac{p_{i}}{N},$$

*where p_i_ is the pixel-specific population count, and N is the total population count falling into a given province.”*

- **Why were verification metrics inconsistent (e.g. CRPS, RMSE and MAE were small but DIC and WAIC were big in Table 1)?**

The apparent inconsistency in the different metrics is due to the fact that they measure different aspects of the model. Whilst CRPS, RMSE and MAE measure absolute deviations from the observed number of cases and are meaningful in isolation, DIC and WAIC are information criteria that assess model goodness of fit, and are meaningful when compared to other models fitted to the same data. In this revised version, we only use the CRPS as a verification metric as this metric is more suited to probabilistic forecasts. The CRPS generalizes the MAE to probabilistic forecasts. We have included this rationale into the manuscript (page , lines 295-297).

*“The predictive ability of each model was evaluated using the continuous rank probability score (CRPS) which generalizes the mean absolute error for the case of probabilistic forecasts. The advantage of the CRPS compared to the MAE or RMSE is that the CPRS does not focus on a specific point of the probability distribution of the forecasts, but on their distribution as a whole. CRPS values were computed in R using the SpecsVerification package [73].*

- **Several explanatory variables (e.g. minimum temperature, maximum temperature, and sea surface temperature anomalies) were likely to be highly correlated (L315-319), and how was this issue resolved?**

Correlation coefficients were high (i.e. above 0.7) only between Tmin_02_ and Tmax_02_ (rho=0.94) which are not included in any of the final models used in the revised version of the manuscript. Also, the main danger of such data redundancy is related to overfitting of the models in question. Here, we used a time series cross-validation approach to penalise overfitted model formulations that did not perform well on out-of-sample datasets. We have added the following text to the **Results section** (page 12, lines 436-441)**:**

*“One major issue with regression models is potential over-fitting which may arise from data redundancy due to high correlation between predictors. The Spearman rank correlation coefficients calculated for all pairs of candidate predictors was less than 0.7, except for between minimum and maximum temperature (rho=0.94). However, these two variables were not present in any of the best performing models.”*

- **Combining all best performing models into a model superensemble led to a lower CRPS and MAE than any of the five competing models, but the RMSE was an exception as the RMSE of the superensemble was higher than two of the five models (L335-337). Why is it the case?**

The difference in performance seems to derive from the fact that whilst for RMSE the errors are squared, for MAE and CRPS the errors scale linearly. In this revised version, however, we decided to use only the CRPS because it is more suited to probabilistic forecasts. The advantage of the CRPS compared to the MAE or RMSE is that the CPRS does not focus on a specific point of the probability distribution of the forecasts, but on their distribution as a whole.

- **Fig 8 shows a range of cost-loss ratios and outbreak thresholds for 42 Vietnamese provinces. How about other 21 provinces as Vietnam has 63 provinces?**

Thank you for highlighting this lack of clarity. In Figure 8 in the previous version of the manuscript we showed only those provinces where the cost-loss analysis shows value at multiple cost-loss ratios. At the time, we decided not to include those provinces where there was no value or there was value for a single cost-loss ratio as the boxes would appear blank. The corresponding section has been largely modified following peer-review suggestions. Consequently, we removed the original figure from the manuscript.

- **It is important to demonstrate that their spatio-temporal superensemble could guide changes to the current practice (L479-84). However, no results were presented on this aspect.**

The system was developed in collaboration with stakeholders and the contents of this manuscript represent our combined inputs. However, the assessment of the operational phase of the project, and its eventual policy impacts have not yet been completed (pages 18, lines 591-596).

*“Our results demonstrate that our spatio-temporal superensemble* ***has the*** ***potential to*** *guide changes to the current practice in dengue control towards a more preventative approach allowing bespoke and targeted public health interventions, and a more efficient allocation of scarce resources. Research on use of the system by stakeholders and its effect on changing dengue control practices is currently ongoing.”*

**Reviewer 2:**

- **I have mixed feelings about this paper. On the one hand, the work is really quite impressive, with a diverse set of top-notch datasets and cutting-edge methods. I found myself nodding away enthusiastically as I read the methods section and text of the results.**

We thank the reviewer for the positive comments on our manuscript.

- **On the other hand, though, the actual results are not very good. The superensemble of forecasts gives effectively the same performance as the better models do individually, as can be seen from table 1 but more clearly from supplementary figure 2. The text therefore feels misleading: yes, the superensemble may be technically better but effectively it was the same and I would have wanted to have seen a more balanced description of that, because this is the sort of project that other countries should be copying and they need the right information to evaluate how complex to aim for.**

Thanks for pointing this out. A more balanced description has been included.

**Results**

*“Averaged across all lead times, the superensemble led to* ***slightly lower CRPS values*** *than each of the competing models, including the baseline (Table 1). However, when stratified by lead time, the superensemble outperformed the competing models at all time leads but only outperformed the baseline at leads of one to three months (Fig 1A). The uncertainty of the forecasts increased with lead time. This is also evident when using the CRPSS for which values above zero (indicating better skill) were observed for the superensemble only for leads one to three months (Fig 1 B). The CRPSS can be interpreted as the relative improvement observed from using a model compared to a reference baseline. Thus, a value of 0.4 would suggest that the values from a given model have an absolute error that is 40% smaller than the absolute error of the baseline model. The CRPSS indicates that at a lead time of one month the superensemble and the competing models outperform the baseline by about 42% whereas at a time lead of six months their absolute error is about 12.5% larger than that of the baseline model.*

*All models showed a tendency to overestimate the predicted number of dengue cases indicated by bias values above zero (Table 1). Bias also increased as the forecast horizon increased suggesting that the tendency to overestimate increases as the forecast horizon increases (Fig 1 C). The model with the smallest bias was the baseline followed by the superensemble. The sharpness of the forecasts deteriorates with lead time (Fig 1 D) as smaller values indicate a better forecast. The superensemble showed slightly worse sharpness than two of the competing models for lead times of four to six months.* **(page 13, lines 451-470)**

*“From February to June, however, the skill of the baseline model is better than that of the superensemble for multiple provinces identified by CRPSS values below zero, particularly during Mach and April.”* **(page 14, lines 488-490)**

*“A ROC curve analysis indicates that using the superensemble with a moving outbreak threshold based on the endemic channel plus two standard deviations resulted in 73% correct predictions. Similarly, using the superensemble with an outbreak threshold based on the 95^th^ percentile led to 72.5% correct predictions. The baseline model had the lowest skill with 61.3% correct predictions (Fig 6). However, using the 95^th^ percentile outbreak threshold resulted in a slightly higher probability of detection (70% of all observed outbreaks) than using the endemic channel plus two standard deviations (68%). The baseline model had the lowest probability of detecting outbreaks (54.5%).”* **(page 15, lines 508-516)**

**Discussion**

*“We demonstrate that the superensemble slightly outperforms the predictive ability of the individual probabilistic models selected from a suite of top performing models at multiple lead times and months of the year in line with previous research [37,44,86,87]. Compared to a baseline model that predicts the same dengue incidence rate for each season and province, the superensemble generated more accurate predictions at lead times of one to three months but not beyond. Model performance was assessed using a range of verification metrics for probabilistic forecasts across time and space. To our knowledge, this is one of the first early warning systems informed by Earth observations to demonstrate skill for prospective year-round dengue prediction in a robust out-of-sample framework. It is also one of the first prototypes for routine dengue early warning at multiple time leads.”* **(pages 17-18, lines 587-597)**

*“Relative to a baseline model, the superensemble made slightly more accurate predictions of the estimated number of dengue cases across most provinces, at lead times of one to three months in agreement with previous studies [17,41,44]. At lead times beyond three months, however, the baseline model made more accurate predictions possibly because incorporating seasonal climate data into predictive models may increase the noise in the data set [44]. Previous research found that climate naïve models may outperform climate-informed models [31,44]. Those studies, however, used different sources of climate and environmental data than our study and were conducted in different settings (i.e. Thailand, Peru, and Puerto Rico) where the effects of climate might be significantly different to those observed in Vietnam. In addition, those studies initialised their prospective forecasts at fixed times of the year whereas our study initialised forecasts at each calendar month providing the model with more opportunities to learn the relationships between dengue incidence and each of the climatic and socio-demographic predictors.”* **(page 18, lines 607-620)**

- **The other reason I say the results are not very good is that the forecast at six months is really poor. Figure S6 illustrates this clearly: in March, the authors are trying to forecast the situation in August, and for HCMC can't even tell if the number of cases will be a few hundred or 20 000. If there is this level of uncertainty, the more honest thing to do would be to report that the forecast accuracy is intolerable at that forecast window and instead limit the forecast to say one to two months into the future. I thought this paper was great until I went to the supplement and had the chance to see directly what the forecasts looked like: they are really not presented transparently within the ms itself.**

Thank you for highlighting this issue. It is true that the uncertainty levels for Ho Chi Minh in the initial version of the manuscript were considerably high at lead times of three to six months. However, this was not the case for any of the other provinces portrayed in the manuscript. The situation seems to have arisen from 1) the configuration of the system which has now be modified thanks to the peer-review process, and 2) the fact that Ho Chi Minh has a much larger interannual variability in dengue cases than any other province in Vietnam. The standard deviation in the annual number of dengue cases is 15,775 which is almost twice that of Ha Noi (9,126), almost three times larger than that of Dak Lak (5,522), and almost four times that of Dong Nai (4,229). Thus, it is expected that the credible intervals for HCM would be larger than for other provinces.

After we made changes to the model structure as suggested by the reviewers, the maximum level of the credible interval of the predictions for the transmission season (i.e. May to October) is considerably lower than when we used the previous methodology (see Figure below). We can observe that while the model overestimates the number of cases in Dak Lak, the observed number of cases for the other provinces are all within the credible interval of the predictions. This forecast was initialised on 10 May 2020 for the period May to October 2020 using data up to 30 April 2020.

We emphasize that the forecasts and their corresponding uncertainty levels are provided to users with a suite of province- and month-specific verification metrics so that they can decide on the level of trust they put on a particular forecast. These metrics include the CRPS, CRPSS, and Brier score. The CRPSS, for example, can be interpreted as the relative improvement observed from using a model compared to a reference baseline. Thus, a value of -0.4 would suggest that the values from a given model have an absolute error that is 40% larger than the absolute error of the baseline model, and so users may decide not to use the forecast and base their decision-making processes on historical seasonal averages instead. Users are also provided with the probability that the number of cases may exceed the moving outbreak threshold defined as the historical month-and province-specific mean plus two standard deviations or the 95^th^ percentile of the historical distribution of dengue cases.

- **My second major concern is about the cost analysis, which is poorly handled and lowers the overall quality of the manuscript. I don't think the equation is correct (surely the optimal decision depends not just on the ratio of cost under action 1 and benefit under action 2 but also the impact of the two actions on the outcome and the costs of action 2 and benefits of action 1), and the statement about always or never preparing for outbreaks being the default raises alarm bells. The decision-making process about interventions for outbreaks of dengue is way more complex than the authors are making it out to be and the analysis here really comes across as the view from an ivory tower. If the authors really want to do a cost-benefit analysis, perhaps because the grant promised it, then it would behove them to do it properly. You have some really great people like Mark Jit at LSHTM, why not tap on them to see how to do a proper CBA? (Probably as a separate paper though.)**

We acknowledge that this analysis does not accurately quantify the complex decision-making process nor the costs associated with outbreak response in the country. Such cost-benefit analysis is the subject of ongoing future work. The analysis in this paper is useful as a screening process to identify areas in which a forecast is most likely to be cost-effective and thus, target our more thorough evaluation studies in the future. We also acknowledge the confusion this section may create, and so have made a number of modifications to de-emphasise the economic aspects of this analysis. In particular, we have re-named the analysis from “relative economic value” to just “relative value” to avoid confusion. This is now re-worded as follows:

**Methods**

***“Relative value of using a forecasting system***

*Unlike other skill measures, the relative value (V) of the forecasts generated by the superensemble depends on requirements set by the user [83]. Typically, V is evaluated in monetary terms and is particularly useful when the probability of occurrence of an adverse event (e.g. a major outbreak) is known. If the probability of occurrence of an outbreak is greater than the ratio of the cost of taking preventive action divided by the loss incurred by not taking action (C/L ratio) and an outbreak occurs, then it will pay off to take action. If the probability of occurrence of an outbreak is lower than the C/L ratio, then it does not pay off to take preventive action. If the probability is equal to the C/L ratio, it does not matter if action is taken or not. Our analysis provides an initial screening process to identify areas where a forecast is most likely to be cost-effective and thus, target of comprehensive economic evaluation studies in the future.*

*Given that data on the cost of taking preventive action, and on the losses incurred by not taking action were unknown to us at the time of writing this manuscript, V was estimated for a range of theoretical C/L ratios following [83] by comparing the mean cost of using the forecasting system for outbreak detection compared to the mean expense incurred by either never preventing outbreaks or, on the contrary, taking preventive action every month of the year. V takes a value of one if the forecast is perfect, and a value of zero if it is no better than the default action plan. If V is negative, it indicates that the forecast is so poor that it would be more cost effective not to use it. The algebraic definition of V is:*

*where E(S) is the theoretical expense incurred by taking preventive action each month or the losses incurred by no taking action at all even when an outbreak occurred, whichever is the cheapest method when not using the forecast; E(A) represents the total cost of the forecast calculated as the cost of type 1 (false positive) and type 2 (false negative) errors plus the cost of acting when an outbreak was predicted and it occurred (true positive); and E(P) indicates the cost incurred with a perfect forecast where outputs are exclusively true positives and true negatives.”* **(page 12, lines 405-431)**

**Results**

***“Relative value***

*An analysis of the relative value of using dengue forecasts generated by the superensemble can be undertaken using the model outputs to guide decisions-making processes. For such analysis, a value index ranging between zero and one is used, with one indicating a perfect forecast [83,84]. The relative value of the forecasts is interpreted in comparison to either never taking preventative action or always taking action.*

*We defined a range of theoretical epidemic thresholds ranging between the 51^st^ to the 99^th^ percentiles of the distribution of dengue cases for the whole time series. Outbreak thresholds were province- and month-specific. Overall, the forecasts generated by the superensemble showed relative economic value in 76% of the provinces one month ahead, 73% of the provinces two months ahead, 68% of them three months ahead and 65% of the provinces four to six months ahead (Fig 9). We note that the superensemble has relative value in areas where dengue is typically endemic such as the central and southern provinces. The superensemble had no relative value in the northern provinces where dengue is typically absent.”* (pages 16-17, lines 543-557)

- **My final major criticism is on the structure of the forecast models themselves. Why are no lags considered for the predictors? Some such as El Nino would expect to see their presence felt a long time after. Even local features could plausibly have an impact longer than one month in the future because of the long latency caused by the cycles of breeding, infection and both incubation periods. You should be able to quickly refute this.**

We have considered the delayed effects of the climatic predictors based on previous research. In particular, we account for the potential delayed effects of temperature, precipitation, specific humidity and sea surface temperature. This is explained in the **Methods** **section** as follows (page 8, lines 268-275):

*“Delayed effects of meteorological factors on dengue were accounted for by incorporating a three-month moving average of temperature, precipitation, specific humidity, and diurnal temperature range (DTR) centred at lag of one month (i.e. each value is given the average of lag zero to a lag of two months). Sea surface temperature anomalies in the Nino region 3.4 were lagged zero to three months based on previous research [19,32,69] and exploratory analyses. No delayed effects were considered for wind speed based on previous research indicating a highest effect of wind at a lag of zero months [70].”*

- **Furthermore, why are the data at such a crude temporal resolution? If the VN MOH has provided an author, can s/he not provide weekly (or even daily! That would be fab!) data, because that would presumably greatly improve the predictive potential.**

It may be possible to obtain weekly or even daily reports of dengue cases across Vietnam. However, weekly data are only available for the period 2017-2020, and daily data for the period 2018-2020, whilst monthly data are available for the period 2002-2020. Also, using weekly or daily data in our models would likely introduce more noise into the models. Moreover, planning in Vietnam is conducted on a monthly basis and stakeholder feedback indicates that their preference is to have monthly estimates of dengue risk. Working with monthly data also minimises the issue of differential reporting delays between different areas. Lastly, intra-monthly variation in the meteorological variables would introduce a considerable amount of noise into the system that may hamper the signal-to-noise ratio required to make accurate predictions.

**A few minor comments that may help revise the manuscript:**

- **Abstract: "the skill of a system using different lead times in a year-round prediction system has not been previously explored" I'm not sure that is strictly true. For instance, Singapore has been doing something like this for a number of years**

We thank you for highlighting this. We have modified the text in the Abstract as follows (page 1):

*“the skill of a system using different lead times in a year-round prediction system has been seldom explored.”*

We have also included the following reference in the introduction:

*@ARTICLE{shi16,*

*author = {Yuan Shi and Xu Liu and Suet-Yheng Kok and Jayanthi Rajarethinam and Shaohong Liang and Grace Yap and Chee-Seng Chong and Kim-Sung Lee and Sharon S. Y. Tan and Christopher Kuan Yew Chin and Andrew Lo and Waiming Kong and Lee Ching Ng and Alex R. Cook},*

*title = {{Three-Month Real-Time Dengue Forecast Models: An Early Warning System for Outbreak Alerts and Policy Decision Support in Singapore}},*

*journal = {Environ Health Perspect},*

*year = {2016},*

*volume = {124},*

*pages = {1369-1375},*

*timestamp = {2017.08.14}*

*}*

- **L13: \textit{Ae.~aegypti} to prevent inter-sentence spacing. You can similarly use this trick after i.e.~ and so on**

Thank you for the suggestion. We have amended all instances as suggested.

- **L10: International standard is to use a space to demarcate thousands from hundreds to avoid ambiguity about decimal point vs decimal comma**

Thank you for the suggestion. We have amended all instances as suggested.

- **L24-32: A bit of repetitive sentence structuring, consider tweaking.**

We have modified the text as suggested to avoid repetition. The amended version is as follows (page 3, lines 28-35):

*“Dengue control in the country primarily involves community engagement and mobilisation to reduce breeding sites, and outdoor low-volume insecticide spraying in the vicinity of reported dengue cases to kill adult mosquitoes [10]. One limitation of dengue control measures is that they are essentially reactive, meaning they take place after cases have occurred. Thus, vector control, dengue diagnosis and reporting all suffer delays which vary across time and space [16]. This situation hinders the timely generation and communication of information on when and where transmission occurs, limiting the ability of health professionals to plan and execute control measures.”*

- **L39-48: These sentences were a bit hard to read. Consider tweaking with some extra words at the start of the sentences like "It is also known that", "In contrast," or changing the pattern a bit like "Biting rates are also affected by xxx" etc, to help with the flow**

We have amended the text as suggested. The amended version reads (pages 3-4, lines 40-59):

*“It is known that temperature regulates the development, biting, reproduction rates, and the spatial distribution of Aedes mosquitoes [20-22]. The titre and replication of dengue viruses within mosquitoes is temperature dependent [20, 23]. For example, rising temperatures increase dengue transmission to an optimum range of 26–29◦C [24], and large diurnal temperature ranges (above 20◦C) reduce transmission and increase mosquito mortality [25]. Mosquito survival is also affected by humidity. Research indicated that Ae. aegypti eggs survive across a wide range of humidity and temperature combinations. However, Ae. albopictus eggs experience high mortality when relative humidity is lower than 95% if temperatures are above 22◦C [26]. Under high humidity conditions (i.e. above 80%), adult mosquitoes survive and remain active by replenishing transient body fluid depletion with plant sugars and host blood [27]. This behaviour is also observed in lower humidity environments although to a lesser extent [27]. The effect of precipitation on dengue is related to the creation or flush away of mosquito breeding sites [28]. The effects of wind speed have been seldom explored in the literature. However, research suggest that wind speed reduces the biting activity of mosquitoes reducing dengue risk [29]. Most of the effects of climate on dengue incidence are delayed by one to two months due to their effect on the life cycle of both mosquitoes and the dengue virus [30]. Thus, the climatic conditions just before the transmission season starts may be indicative of dengue incidence in the following dengue transmission season [31, 32].”*

- **L42: bad latex: $>$ instead of > and either \degree, \textdegree (depending on packages) or $^{\circ}$**

Thank you for the suggestions. We amended the LaTeX code as suggested.

- **L71: What is fundamentally wrong with testing a model on year 2015 after training it on 2016 (say)? Does it not still give a valid approximation of out of sample performance?**

While block cross-validation (i.e. leave-one-year-out) would also be a valid approach to obtain an approximation of out-of-sample error, it has the disadvantage that if a pattern was present in the data at a date later than that we aim to forecast but not prior to it, the model would learn such a pattern and potential bias our estimates. We believe that our approach is better suited to the goal of the project to provide prospective forecasts.

- **L81: Good point, perhaps worth elaborating on the difficulties of implementing such a system not in a research environment**

We have modified the text as follows (page 4, lines 88-92):

*“In some cases, systems are designed exclusively for research purposes in isolation from relevant stakeholders who may become potential users. This situation is partly due to the difficulties related to the integration of these systems into existing public health procedures, lack of technical expertise, and occasional lack of political will.”*

- **L102: $<$ not <**

We have amended the LaTeX code as suggested.

- **L129: Possible to show time series of the data for a few provinces?**

S1 Fig. shows the time series of dengue cases for all provinces over the study period.

- **L142: Perhaps my physics is letting me down but isn't kg kg^{-1} unitless?**

You are correct in that kg/kg is unitless though specific humidity may also be defined as a ratio of masses. To avoid confusion, we now refer to specific humidity as “dimensionless”

- **L152-160: Do I read correctly that you used two different datasets to create population data? How were these harmonised? If not, please clarify and remedy my confusion!**

The SEDAC population data although gridded, has identical values at each pixel falling within a given administrative district (i.e. admin 2 level). Consequently, it could not be used for the computation of population weighted averages. As a compromise, we used WorldPop estimates for which population estimates vary per pixel. At the moment, both sources of population data are used. This is explained in the manuscript as follows (page 7; lines 202-210):

*“Climate data were aggregated at the province level using population weighted averages for each month. The SEDAC population data although gridded, had identical values at each pixel falling within a given administrative district (i.e. admin2 level). SEDAC data could not be used for the computation of population weighted averages as each grid cell within an administrative district would carry the same weight. As a compromise, we used WorldPop [64] estimates for which population estimates vary per pixel. Population data from the WorldPop project [64] at a 100 m2spatial resolution was used to calculate annual gridded weights for each province using the rasterstats module in Python 3.6 [63].”*

- **Eqn1: Should Y be Y_{i,t-1}?**

Thank you for spotting this typo. We have amended equation 1.

- **Should the sum be enclosed in parenthesis to prevent ambiguity about the random effects?**

We believe the parenthesis are not required in this case.

- **What happens when Y=0?**

We believe the question refers to what happens when Y=0 and we compute the logarithm of the observed number of dengue cases in the previous month. To avoid getting infinite values, we used the logarithm of the number of dengue cases plus one lagged one month. We have clarified this in the text as follows (page 8, lines 254-255):

*“log(Y_i,t-1_) is the logarithm of the observed number of dengue cases in the previous month plus one with an auto-regressive parameter ρ.”*

- **L205: missing closing parenthesis**

We have incorporated the missing bracket.

- **Eqn 5: \exp \max**

Thank you for the suggestion. However, in the amended version of the paper we have removed this equation.

- **T1: Interesting that so little benefit accrues from the superensemble compared to simpler models. The text hints that the superensemble is substantially better**

We have modified the text to make a more balanced description of the results (page 13, lines 451-470)

*“Averaged across all lead times, the superensemble led to* ***slightly lower CRPS values*** *than each of the competing models, including the baseline (Table 1). However, when stratified by lead time, the superensemble outperformed the competing models at all time leads but only outperformed the baseline at leads of one to three months (Fig 1A). The uncertainty of the forecasts increased with lead time. This is also evident when using the CRPSS for which values above zero (indicating better skill) were observed for the superensemble only for leads one to three months (Fig 1 B). The CRPSS can be interpreted as the relative improvement observed from using a model compared to a reference baseline. Thus, a value of 0.4 would suggest that the values from a given model have an absolute error that is 40% smaller than the absolute error of the baseline model. The CRPSS indicates that at a lead time of one month the superensemble and the competing models outperform the baseline by about 42% whereas at a time lead of six months their absolute error is about 12.5% larger than that of the baseline model.*

*All models showed a tendency to overestimate the predicted number of dengue cases indicated by bias values above zero (Table 1). Bias also increased as the forecast horizon increased suggesting that the tendency to overestimate increases as the forecast horizon increases (Fig 1 C). The model with the smallest bias was the baseline followed by the superensemble. The sharpness of the forecasts deteriorates with lead time (Fig 1 D) as smaller values indicate a better forecast. The superensemble showed slightly worse sharpness than two of the competing models for lead times of four to six months.”*

- **L367: Some of this subsection should perhaps go in the methods section?**

We have moved lines 367-379 to the Methods section (page 11, lines 377-387):

*“****Outbreak detection evaluation***

*The skill of the model for outbreak detection was evaluated using the Brier score [81]. For the Brier score, smaller values indicate better skill. Four moving outbreak thresholds were defined to evaluate the skill of the superensemble for outbreak prediction: i) the endemic channel plus one standard deviation, ii) the endemic channel plus two standard deviations, iii) the 75^th^ percentile of the distribution of dengue cases per month, and iv) the 95^th^ percentile of the distribution of dengue cases per month. The endemic channel was calculated as the number of dengue cases per month and per province over the previous five years in agreement with current practice at the Vietnamese Ministry of Health. The 75^th^ and 95^th^ percentiles were calculated over the whole observational period at each time step. We then calculated the probability of exceeding the moving outbreak threshold based on the posterior distribution of the predicted number of cases.”*

- **L407/8: missing spaces**

We have now added the missing spaces.

- **L416: The proposed decision support tool is… a bit weak, isn't it? Aesthetically it's ugly, but the uncertainty in the predictions seemingly makes the forecast useless.**

We have modified the colour palette to make the figure more appealing. We also modified the *Y* axis to indicate the incidence rate per 100 000 people instead of the crude number of dengue cases to allow a more balanced comparison between provinces. We also made changes to the model structure as mentioned previously, and it is noted that the upper bound of the credible intervals of the predictions is considerably lower than that in the previous version of the manuscript. We would like to emphasize that stakeholder feedback suggests that users see benefit in gaining access to such long-term predictions despite the large uncertainties as they allow them plan for potential outbreaks. Even knowing that predictions are uncertain beyond a specific timeline is a valuable output as it will allow stakeholders to make contingency plans that should be re-visited at a specific timepoint. Note, the forecasting system provides the predicted number of cases/incidence along with a suite of verification metrics and probabilities, to allow users to make informed decisions.

- **T2: Why is Moderate defined as being up to the 75th percentile AND mean+1SD? Mean+1SD should not be the 75th percentile.**

Thank you for highlighting this issue. We have modified Table 2 to make it clearer (page 15)t

- **L515: What is 95 per cent percentile? Is it the same as the 95th percentile?**

Yes, we referred to the 95^th^ percentile. We have amended the text in accordance.

- **F1: Not very informative. Can I propose you omit the islands (and just add a comment in the legend that islands in the East Sea are omitted for space). Then you can stack the maps in a 6x2 arrangement, making them all bigger and reducing the white space between them. At present you can hardly make out the changes in colour around HCMC and Vinh.**

The islands have been kept in the maps at the request from the General Department of Preventive Medicine, Hanoi.

**Reviewer 3:**

**Overview:**

- **In this manuscript the authors present and evaluate ensemble forecasts of monthly dengue case numbers in each province of Vietnam for 2002-2019, which incorporate a variety of demographic, land-cover, and climate data. These forecasts are evaluated for lead times of 1-6 months --- looking much further ahead than many existing dengue forecasting studies. The evaluation includes a cost-loss analysis of whether action taken in response to the projected outbreak probability would be beneficial. I found this to be an interesting and well-designed study, and the prospect of further evaluation of this forecasting system is exciting.**

We thank the reviewer for the very positive evaluation of our manuscript.

**Major comments**

- **Regarding the "Modelling approach" section on page 6, I understand that
  there were 7 seasonal climate data sets (mean monthly values were used): Minimum temperature; Maximum temperature; Daily precipitation; Specific humidity; Diurnal temperature range; Wind speed; and Sea surface temperature anomalies for the Niño 3.4 Region. So, 128 different models were considered to cover all possible combinations of these 7 data sets. But this only became apparent after reading the first few sentences of the results section, where this is explained to the reader. I would have found it quite helpful to instead include these remarks in the "Modelling approach" section.**

We thank you for highlighting this issue. We have incorporated the following text to the **Model Selection** sub-section: (page 9, lines 291, 292)

*“Thus, we tested 128 unique model specifications across 114 forecast months (i.e. January 2007 to December 2016).”*

- **"Model selection" section (page 6): "The testing set comprised the climate hindcast data for the six months immediately after the last observation in the training set for each geographical area." I apologize if I've missed an obvious point here, but why were hindcasts used instead of actual data for this period? Is the intent that in an operational system they would be replaced by climate forecasts from the same source (Copernicus Climate Data Store)?**

As you correctly inferred, the use of climate hindcasts referred to in the Model Selection subsection were used to simulate what would have occurred should the system had been operational over the period 2007 to 2016. We have clarified this in the text as follows (page 9, lines 293-296):

*“The testing set comprised the climate hindcast data for the six months immediately after the last observation in the training set for each geographical area. Seasonal climate hindcast data were used to simulate the behaviour of an operational system over the period 2007 to 2016.”*

- **"Accounting for autocorrelation in disease transmission" section (page 7): *"To generate dengue forecasts one month ahead, log(Y_i, t−1) for time t + 1 corresponds to the logarithm of the number of dengue cases at time t. To generate the forecasts two months ahead we first fitted a climate naïve model ..."* It isn't clear to me why a climate-naive model was used here. Since the testing set included climate hindcast data for the six months after the last observation (see previous comment) then why couldn't the climate-informed model be used to predict the number of dengue cases for time t + 1? And at the start of the Results section (page 9), the authors write: *"Predictions up to six months ahead were made at each time step starting in January 2007 using seasonal climate hindcasts (retrospective forecasts) data ..."***

The rationale for using a climate naïve model for the computation of dengue predictions was to be able to evaluate the different models under similar conditions. By using a climate naïve model, we ensured that dengue counts at times *t+1 … t+6* were the same for all competing models. We have clarified this in the new section ***Prospective lagged dengue cases*** as follows (page 10, lines 328-348):

*“One complication of accounting for the number of cases in the previous month in an operational system is that log(Y_t-1_) in the temporal window of the forecast can only be known up to time t+1. More specifically, to generate dengue forecasts one month ahead, log(Y_i,t-1_) for time t+1 corresponds to the logarithm of the number of dengue cases at time t. We used a climate naïve model to estimate the log(Y_i,t-1_) for time t+1 to time t+6 to ensure that the number of cases in the previous month were exactly the same for all competing models. Thus, reconstructed lagged dengue time series was exactly the same for all models.*

*To generate the forecasts two months ahead we fitted the climate naïve model with the following specification:*

*with a as the intercept; log(P_i,a[t]_) as the population at risk in province i at time a[t], included as an offset; log(Y_i,t-1_) is the logarithm of the observed dengue cases plus one in the previous month with regression coefficient ρ; γ_i,a[t]_ as province-specific unstructured yearly random effect; δ_i,m[t]_ as province-specific structured monthly random effect with an AR1 auto-correlation term; and u_i_ and ν_i_ as province-specific structured and unstructured random effects.*

*We then predicted the number of dengue cases for time t+1. The logarithm of the predicted number of cases at time t+1 was then used as log(Y_i,t-1_) for time t+2. We repeated these steps for each lead time in the forecast until time t+6. Once the reconstructed lagged dengue time series was complete, we fitted all competing models as indicated in the Model Selection section.”*

- **"Model superensemble" section (page 8): "A new set of superensemble weights were computed each time a forecast was issued using training data from all previous years." Can the authors comment on how these weights evolved over time? Was the same model consistently the highest-weighted over all of the forecast months? Presumably in an operation system it would be highly desirable if the model weights were stable from one month to the next? I suppose this could be extended to allowing seasonal variation (i.e., month-to-month) if different models are understood to perform best in different calendar months. It would be great to see, e.g., a supplementary figure that shows how the weights
  evolved.**

We have generated S2 Fig. where we show the seasonal variation in the weights assigned to each of the model. A description of the figure is provided in the Results section (page 13, lines 446-450)

*“There was strong seasonal variation in the weights assigned to each model (S3 Fig). All competing models had similar weights across the whole evaluation period (Jan 2007 to December 2016), whereas the baseline model had the lowest weights. We note that the weights assigned to the baseline model gradually decreased with time.”*

- **"Model superensemble" section (page 8): "In addition, we evaluated the skill of the obtained forecasts using the continuous rank probability skill score (CRPSS). CRPSS is defined as: ..." It appears that there should be further text after equation (6), since it ends with a trailing comma, but instead the section ends. Is there missing text, or should the comma be removed?**

We apologise for the confusion. We have now completed the following sentence.

- **Results section (page 9): how did the authors decide to include precisely 5 models in the superensemble? Was there a clear divide in performance between the top 5 models and the remaining 123 models?**

In the previous version of the manuscript we had selected the two best performing models for each of the five verification metrics used. This approach led to five models because some of the best performing models were the same for more than one verification metric. In the current version of the manuscript, we also select five models based on the CRPS alone because there was a clear divide between these and the rest of the models. The models selected for this revised version all have a mean CRPS below 90 cases.

- **And were the authors surprised that none of the models in the superensemble included daily precipitation as a seasonal climate predictor?**

This was indeed a surprising result. However, we are working on an improved version of the system and preliminary results suggest that other hydrological variables may improve the predictive ability of the superensemble.

- **Results section (page 10): "The predictive ability of the [superensemble] model also varied with the month of the year (Figure 1). Overall, larger discrepancies between observed and predicted values were observed between July and December, when typically, more cases are reported."**

Thank you for pointing out the need for specifying we are referring to the superensemble. We have replaced the word “*model*” with “*superensemble*” in the amended version of the manuscript.

- **Did the authors consider evaluating the models using any relative error measures, in addition to the chosen absolute error measures? This is an important consideration for infectious diseases epidemic forecasts since an error of, say, 10 cases has very different significance when the expected number is 20 cases or 20,000 cases. As the authors note: "From August to November the skill is reduced for selected provinces in the central and southern regions characterized by larger dengue incidence variability." This might be primarily influenced by the choice of absolute error measures rather than being intrinsic to the models themselves.**

We considered the use of the relative absolute error and the relative squared error in our computations using the *Metrics* R package. However, many provinces had counts of zero cases at multiple time steps. Consequently, the corresponding results equalled infinite values.

- **Results section (page 10): "S5 Fig. shows the observed and posterior predictive mean dengue cases across the 63 provinces computed one month ahead using the model superensemble. The model superensemble is able to reproduce the spatiotemporal dynamics of dengue fever with reasonable skill although predictions tend to underestimate the number of cases particularly during large outbreaks." This observation made me wonder whether the model fitting may not have selected appropriate values for the dispersion parameters. The authors note (top of page 6) that models were fitted "using a negative binomial specification to account for potential over-dispersion in the data" and that "penalising complexity priors were assumed for [...] for both the dispersion parameters and the precision for all random effects ". What prior distribution was used for the over-dispersion parameters, and what were the posterior distributions when the models were fitted? I couldn't see this mentioned in the manuscript text, nor am I able to identify a location in the provided code or supplementary materials where this is defined. In the provided file "04_Fit_models.R" there is the following code:

  ```
  mu1 <- lead6$fitted.mean
  E1 <- (lead6$dengue_cases - mu1) / sqrt(mu1)
  N <- nrow(lead6)
  p <- 6 # Max number coef
  dispersion <- sum(E1^2, na.rm=TRUE) / (N - p)
  dispersion <- data.table(date=j, model="ensmeble", disper=dispersion)
  ```
  But this table doesn't seem to be used anywhere in any of the provided
  scripts, so I gather this it is reporting an output statistic.**

We did not explicitly specify a prior distribution for the dispersion parameter (φ). We used the default *pc.gamma* specification in the INLA package which assumes a Gamma distribution with values of *φ* within the following interval 1-5668.2. This has been incorporated to the manuscript in page 8, lines 279-281.

*“We used the default pc.gamma specification in the INLA R package [72] which assumes a Gamma distribution with values of φ within the interval s1-5668.2.”*

- **Discussion, page 15: "Relative to a baseline seasonal predictive model (see Methods and Materials), the superensemble made, on average, more accurate predictions across most provinces, and for most of the year in agreement with previous studies [16, 39]. This observation, however, conflicts with the results obtained by [30, 42] for whom climate-naı̈ve models had better skill than seasonal-climate-informed ones." Are there any obvious reasons for this apparent disagreement? Did the studies in refs 30 and 42 make use of different data sources, different evaluation metrics, etc? And can the authors make any general remarks about whether the superensemble forecasts outperformed the climate-naive models in refs 30 and 42? I appreciate there are many reasons why a direct comparison may not be feasible, and if that's the case then could the authors please make this clear in the text so that reader isn't left wondering?**

There are many reasons as to why there may be differences in the results of references 30 and 42 and our results. First, both papers used different sources of climate and environmental data than our study. Second, both studies were conducted in different countries (i.e. Thailand, Peru, and Puerto Rico) where the climate signal may be significantly different to that observed in Vietnam. Third, Lauer et al., initialised their prospective forecasts in April each year for the period April to December. Similarly, Johansson et al., defined a date after which no more data could be incorporated into the training set. Conversely, our study initialised forecasts at each calendar month for the following six months and updates the training set at each time point. We have incorporated this into the Discussion (page 18, lines 607-620)*.*

*“Relative to a baseline model, the superensemble made slightly more accurate predictions of the estimated number of dengue cases across most provinces, at lead times of one to three months in agreement with previous studies [17,41,44]. At lead times beyond three months, however, the baseline model made more accurate predictions possibly because incorporating seasonal climate data into predictive models may increase the noise in the data set [44]. Previous research found that climate naïve models may outperform climate-informed models [31,44]. Those studies, however, used different sources of climate and environmental data than our study and were conducted in different settings (i.e. Thailand, Peru, and Puerto Rico) where the effects of climate might be significantly different to those observed in Vietnam. In addition, those studies initialised their prospective forecasts at fixed times of the year whereas our study initialised forecasts at each calendar month providing the model with more opportunities to learn the relationships between dengue incidence and each of the climatic and socio-demographic predictors.”*

- **Discussion, page 15: "Third, our computations of dengue risk do not take into consideration uncertainties due to the potential under- or misreporting of dengue cases. Consequently, our model superensemble forecasts may underestimate the real number of cases occurring at any given time." That's a good point to make. Do the authors have any knowledge about whether case ascertainment is likely to have changed substantially over the period covered by this study (2002-2019)?**

We have incorporated the following text in the **Dengue Surveillance Data** **sub-section** to clarify how suspected and confirmed cases are reported in the country and to emphasize that the criteria have not changed over the study period (lines 160-171).

*“According to the national guidelines on dengue surveillance, suspected cases are defined as people living in or coming from endemic areas or from areas that have had dengue outbreak foci over the previous 14 days with manifestation of sudden high fever lasting for two to seven days, and with at least two of the following signs: hemorrhagic manifestation (positive tourniquet test, hypodermic petechia or ecchymosis, stump hemorrhage or nasal hemorrhage), headache, anorexia, nausea, vomiting, skin flush, rash, muscular, joint and orbital pains, writhe, unconsciousness, pain in liver area or pain when pressing on the liver area, hepatomegaly, thrombocytopenia, or increased hematocrit. Confirmed cases correspond to those confirmed by laboratory tests using Mac - Elisa, PCR, and NS1, or virus isolation. Over the period 2002-2019, there have been no changes in the classification of suspected and confirmed dengue cases in Vietnam.”*

**Minor comments**

- **Top of page 3, there is an unexpected character: "Large diurnal temperature ranges (¿ 20°C) ...". Top of page 4, there is an unexpected character: "In areas where dengue incidence is typically low (e.g., ¡ 10 cases per month) ..."**

Thank you for highlighting these typos. We have corrected all instances related to the use of “<” and “>” symbols.

- **Figure S7: consider amending the legend to read "No Economic Value" and "Economic Value", so that it is explicit what "value" is being considered.**

Thank you for the suggestion. We have modified the figure and incorporated the legends “Relative value” and “No relative value” (page 20, Fig 9).

**Reviewer 4:** 

- **This is an interesting paper on an important subject, the development of early warning systems for vector-borne diseases, focusing here on dengue in Vietnam. While the general approach appears reasonable there are a number of clarifications and amendments required. Specific comments are given below.**

Thank you for the positive comments.

- **The model predictions presented by the authors seem useful but they are currently somewhat of a black box. In particular, there are two directions for improving the presentation of the results. The first is based upon standard prequential theory of proper scoring rules and the paper would improve if the authors would decompose the overall prediction error for the different scoring rules into calibration and sharpness, the predictive measures corresponding to bias and variance**

We thank the reviewer for this important suggestion. We have incorporated measures of bias and sharpness on Table 1 in the amended version.

- **In addition, it would be very useful for this work, as well as future developments, to see the individual contribution to the predictive ability of the different components. For example, are the climate forecasts adding substantially to the predictive ability or the earth observation data correspond to the best part of the ensemble's predictive power?**

We agree that it would be useful to compare the results of the model using Earth observations to those using forecasts and we will consider that analysis for future developments of the system.

- **Also, how accurate climate forecasts are the predictors of seasonal climate, i.e. how good are these forecasts on their own right? This will be illuminating and could also guide the authors' future efforts in improving this work. It will also shed some light upon the paper's drawbacks, since absence of disease control and related data appears rather important, at least a-priori. In addition, it will lead to a discussion on issues of model mis-specification, since moderate predictive ability of a covariate (like seasonal climate) but reasonable prediction of a function thereof (such as disease incidence) suggests issues of potential model mis-specification.**

A skill analysis demonstrates that their skill of the seasonal climate forecasts is good. As can be observed from the plots below (S2 Fig in the manuscript), the discrepancies (measured using the continuous rank probability score, CRPS) between observed and predicted values are considerably small for all variables and at all lead times.

- **A vital aspect of this work is the predictive ability of this approach and the authors claim that this is superior to the competition. However, as far as can be seen in this paper, the comparison is not made with a number of state-of-the-art alternatives, but with a baseline model which is a sub-case of the model class used in the paper. Is that an accurate description? If so, are the model's conclusions a fair representation of the paper's findings? This should be clarified and amended as appropriate.**

We acknowledge that while we did not explicitly state that our superensemble was better than any specific alternative algorithm, some of the tone of the manuscript may have given that impression. Our primary approach was just to outperform previous practice in Vietnam where no forecast system was used to guide decision-making processes. We have amended the Conclusions section to reflect this (page 19, lines 665-680).

*“We have demonstrated that a superensemble of probabilistic dengue models, formulated using Earth observations and driven by seasonal climate forecasts* and a reconstructed/prospective estimate of lagged dengue cases*, is useful to generate probabilistic predictions of dengue risk across Vietnam at multiple lead times and months of the year. We acknowledge there are alternative state-of-the-art approaches to* *dengue forecasting that may be equally as effective as the one presented* *here. However, our aim was to develop a system that outperformed previous* *practice in Vietnam where no forecasting system was used to guide decision-making processes. A theoretical analysis showed that compared to not using a forecasting system, the superensemble has relative value across most of the country, suggesting that it has potential for guiding decision-making processes. The dengue forecasting system presented here has been rolled out across Vietnam and could be tailored for other dengue endemic countries. Further work may include investigating the feasibility of producing probabilistic forecasts with sufficient skill at the district or commune levels and a comparison with other statistical and mechanistic approaches.”*

- **An important clarification should be made with respect to terminology. Is model superensemble an alternative name for Bayesian model averaging (BMA)? If so, this should be clarified. Incidentally, BMA essentially started in the fifties with the work of Savage, Lindley and Good so appropriate referencing could be added. Non-Bayesian (e.g. AIC-based) model weights are also commonly used, including in ecology where the Burnham and Anderson's 2002 book on multi-model inference is highly influential. In relation to the above comment, please clarify the definition of the ML weights used in equation 5. The DIC weights are often used as a Bayesian equivalent of the AIC-based ones. However, the most commonly used weights are BIC-based, essentially since they agree asymptotically with standard Bayesian theory. However, ML weights are non-standard and the authors will need to specify how are they calculated and give a reference to their theoretical justification.**

Initially we used Bayesian model averaging for the generation of our superensemble. Thus, it would be fair to say that in the previous manuscript the superensemble was an alternative name for it. For this amended version however, we use a different approach using proper scoring rules, particularly the continuous rank probability score (CRPS), following Yao, et al., (2018), Bayesian Analysis 13(3): 917–1003. This new approach has resulted in lower error estimates than using BMA and ML weights. In the amended version, weights are calculated following Kang (1986):

$$w_{i}=\frac{{(V}_{i})^{-1}}{\sum_{j=1}^{n} (V_{j})^{-1}},$$

where V is the variance (square of the CRPS) of the i^th^ forecast errors.

- **The model class that this work is based upon seems reasonable. However, some details need to be added, including the specific parameterisation of the negative binomial distribution and the definition of mu (is it the mean of the log data?)**

The parameter μ is not the mean of log data but a re-parametrization of the mean so that:

Y ~ NBinom(μ, φ),  y >=0

where E[Y] = μ, and var(Y) = μ (1 + μ / φ), and φ is the dispersion parameter.

This is now explained in the methods (page 8, equation 3, line 234)

**Reviewer 5:**

- **The manuscript presents a Bayesian spatio-temporal model for Dengue counts, which incorporates weather and land-cover variables. The five subsets of covariates that produced the best one-to-six-months forecasts during the period 2007-2016 were combined via Bayesian model averaging. This superensemble outperformed the ensemble members in terms of both CRPS and point forecasts (RMSE, MAE). It was eventually used to generate probabilistic forecasts for the 6 months ahead of April 2020. Cooperation with key stakeholders from public health institutions strengthens the paper and supports transferring scientific progress into practice. The paper is well written overall, but I have several questions and remarks about details of the model and how forecasts are generated.**

Thank you for your kind comments.

**Major comments**

- **An AR1 prior is used for \delta_{i,m[t]}. Shouldn't the model assume a cyclic effect, i.e., have January conditioned on December?**

We tested using a cyclic random effect for month of the year. However, the accuracy of the predictions decreased. Consequently, we decided to keep the AR1 specification.

- **Eq (1): What is the motivation behind scaling past counts by the population, i.e., having the terms (1-\rho) log(P) + \rho log(Y)? I think I haven't seen this in related models before. Did explorative analyses indicate a better fit when incorporating past incidence instead of past counts?**

The models used the logarithm of the past counts of dengue cases. We have amended this in the corresponding section, as well as the model equation.

- **The model fit and forecasts could probably be improved by allowing for a region-specific effect of log(Y_{i,t-1}), i.e., region-specific force of infection \rho_i. Scaling by the population would no longer be necessary and the model would more adequately capture regional variation in unobserved important determinants of disease spread. A recent example of a related model with region-specific force of infection** **is** [**https://doi.org/10.1002/sim.8390**](https://doi.org/10.1002/sim.8390)**.**

We tested whether including a region-specific ρ would result in better predictions. However, that specification led to convergence issues at multiple time steps and so we decided to keep the specification presented in the amended version of the manuscript.

- **I was really confused by how k-step-ahead (k>1) forecasts are generated. The manuscript intends to provide superensemble forecasts of regional dengue incidence up to 6 months ahead *using seasonal climate forecasts*, but then a *climate naive* model is used to generate forecasts for t+2 to t+6? Why make the (impressive!) effort of collecting climate data and forecasts if they are not used to inform probabilistic forecasts?**

The generation of forecasts one to six months ahead is conducted in two stages. In the first stage, we use a climate-naïve model to obtain estimates of the number of dengue cases at times t+1 to t+6 so that we can compute the logarithm of the number of dengue cases lagged one month. Note, these values may only be known up to t+1 but not beyond that point. We used a climate naïve model to predict the prospective lagged dengue cases, to help inform the models formulated using climate information in stage 2. In the second stage, we use the data including the newly computed logarithm of the lagged number of dengue cases to generate predictions for one to six months ahead using climate-informed models. We have clarified this in the text as follows (page 10, lines 328-348):

*“One complication of accounting for the number of cases in the previous month in an operational system is that log(Y_t-1_) in the temporal window of the forecast can only be known up to time t+1. More specifically, to generate dengue forecasts one month ahead, log(Y_i,t-1_) for time t+1 corresponds to the logarithm of the number of dengue cases at time t. We used a climate naïve model to estimate the log(Y_i,t-1_) for time t+1 to time t+6 to ensure that the number of cases in the previous month were exactly the same for all competing models. Thus, reconstructed lagged dengue time series was exactly the same for all models.*

*To generate the forecasts two months ahead we fitted the climate naïve model with the following specification:*

*with a as the intercept; log(P_i,a[t]_) as the population at risk in province i at time a[t], included as an offset; log(Y_i,t-1_) is the logarithm of the observed dengue cases plus one in the previous month with regression coefficient ρ; γ_i,a[t]_ as province-specific unstructured yearly random effect; δ_i,m[t]_ as province-specific structured monthly random effect with an AR1 auto-correlation term; and u_i_ and ν_i_ as province-specific structured and unstructured random effects.*

*We then predicted the number of dengue cases for time t+1. The logarithm of the predicted number of cases at time t+1 was then used as log(Y_i,t-1_) for time t+2. We repeated these steps for each lead time in the forecast until time t+6. Once the reconstructed lagged dengue time series was complete, we fitted all competing models as indicated in the Model Selection section.”*

- **Furthermore, how is the forecast distribution generated? Are these simple negative binomial forecasts conditioning on the *point* forecast for y_{t+1}, ignoring the uncertainty in that forecast? I'm not sure if this counts as "properly reflect forecast uncertainty" (discussion). Maybe simulation-based path forecasts should be used instead?**

At each time step, we make predictions for each of the 28 ensemble members that comprise the seasonal climate forecast. Thus, we do incorporate the uncertainty in the climate forecasts in our computations of dengue risk. In addition, we generate one thousand samples from the posterior distribution of the point forecasts to reflect forecast uncertainty. We now mention this in the Discussion (page 17, lines 559-565):

*“This study details a probabilistic dengue early warning system based on a model superensemble, formulated using Earth observations, and driven by seasonal climate forecasts. The system is able to generate accurate probabilistic forecasts of dengue metrics that have the potential for guiding policy- and decision-making processes in Vietnam. We make predictions for each of the 28 ensemble members of the seasonal climate forecast, and generate one thousand samples from the posterior distribution of the predicted number of cases to reflect forecast uncertainty.”*

- **Eq (3): I think it may be useful to note that the baseline model isn't such a simple model after all. The province-specific and time-varying random effects will compensate for much of the missing X variables. I think a more important "feature" of the baseline model is the missing autoregressive term. This omission should also be kept in mind when discussing performance relative to this model around p. 15, l. 504. I would expect a non-dynamic model to perform particularly well in regions with only sporadic dengue cases. Please also report the performance of the baseline model in Table 1. Can we conclude from Figure 1 that it would make sense to include a non-dynamic baseline model in the ensemble? As Reich et al (2019) put it: "building ensemble models that capitalize on the strengths of a diverse set of individual component models will be critical to improving accuracy and consistency of models in all infectious disease forecasting settings." (**[**https://doi.org/10.1073/pnas.1812594116**](https://protect-eu.mimecast.com/s/RWp8CB6q0U72kEzcgDGFn?domain=doi.org)**)**

Thank you for this very useful suggestion. In this revised version, we have incorporated a non-dynamic baseline model as in Reich et al (2019) to our superensemble. We also compare the performance of the superensemble against this new baseline. The new baseline model forecasts the same historical seasonal average of the dengue incidence rate every month. We report both the performance of the superensemble and the baseline model in multiple sections of the manuscript. The definition of the baseline model is reported in the Methods section (page 10, lines 350-356).

*“We developed a historical baseline model, which forecasts the same seasonal average of dengue incidence every month at each model run following [17,33,77] to compare all models to a common reference. The baseline model is specified as follows:*

*where α is the intercept; log(P_i,a[t]_) is the population at risk in province i at time a[t], included as an offset; γ_i,a[t]_ indicate province-specific unstructured yearly random effect; δ_i,m[t]_ as province-specific monthly structured random effect with an AR1 auto-correlation term; and u_i_ and ν_i_ as province-specific structured and unstructured random effects.”*

- **One model should really be added to prove the usefulness of the superensemble for forecasting: Does the superensemble outperform a model which contains all of the covariates of models 1 to 5 combined? If so I'm willing to believe that "we demonstrate that using a model superensemble results in better forecasts than using individual models" (discussion).**

In this revised version, Model 1 includes all of the covariates in all of the other models combined. The superensemble outperforms all of these models at all time leads as can be seen from the following figure. This figure has been incorporated to the manuscript in page 13.

- **Table 1: Please report which lags have been selected for the climate predictors in the different models. I think this could be an interesting result by itself.**

We have specified the lags used for the climate predictors in Table 1.

- **The ROC analysis was primarily used to compare AUC values across different forecast horizons (Figure 5), which is not very interesting. It seems more relevant to compare how well the superensemble can forecast the four different outbreak indicators (there does not seem to be much difference but it is difficult to compare across plots). The text also says that an optimal cut-off value for the probability is defined to maximize sensitivity and specificity (which is no clear criterion since you cannot maximize both), but I cannot find the resulting cut-offs.**

We have replaced Figure 5 in the previous manuscript with Figure 6 (below) where we show a ROC curve analysis indicating the proportion of correct and incorrect predictions the superensemble and the baseline model had over the verification period.

**Minor comments**

- **Strong underreporting:**
  - **Intro (first two paragraphs): The question arises why there is such strong underreporting.**
  - **Does dengue seldom require treatment?**
  - **The intro should probably mention common symptoms of dengue infection.**

We have incorporated the following into the Introduction (page 3, lines 5-18):

*“Dengue infection is4characterised by flu-like symptoms that may include sudden intermittent high fever, retro-orbital pain, muscle and joint pain, severe headache, and widespread red skin rash. Its treatment typically includes the supportive care of symptoms. There is no specific antiviral treatment for dengue, and efforts to control transmission focus on controlling vector populations [6]. Dengue prevention relies on mosquito control measures which are primarily insecticide-based [7]. The increasing resistance to insecticides highlights the need for targeted and effective interventions [8].*

*Vietnam is particularly affected by dengue with an estimated burden of about two million yearly infections [5, 9], although, on average, only 95 000 cases have been reported annually to the Ministry of Health over the period 2002–2020. Under-reporting may be due to two main factors. First, dengue surveillance in Vietnam is mostly passive, relying on clinical cases reported by patients seeking healthcare [10]. Second, up to 80% of the cases may be asymptomatic or minimally symptomatic and will likely not seek healthcare [11].”*

- **At the end, the discussion briefly mentions that the model only considers *reported* disease incidence. Thus, routine forecasting relies on stable reporting rates. If there were efforts to reduce underreporting at some point, endemic channels or quantiles based on historical incidence wouldn't make sense any more. Are there any such efforts in Vietnam? How could forecasts integrate such a (gradual) change?**

The dengue reporting network has been consistent over the past 20 years and mainly relies on clinical cases reported by patients seeking healthcare. At the time of this response, there are no major changes scheduled for moving towards an active surveillance system. Should there be any changes, they could be incorporated into the system by using Boolean variables to indicate the times before and after the implementation of such strategies.

- **p. 3, l. 82: "deterministic models" are mixed up with "point forecasts" here. ARIMA or Poisson regression used in the referenced papers are *not* deterministic. The point is that you would like to quantify the uncertainty of your forecasts in addition to your best (point) estimate of future disease spread.**

Thank you for highlighting this. We have made the change to point forecasts.

- **p. 4, l. 102: This sounds like CUSUM methods should be considered, see e.g.,** [**https://doi.org/10.1016/j.prevetmed.2009.05.017**](https://protect-eu.mimecast.com/s/JqeYCD8vnC5GWgDh9fLOQ?domain=doi.org) **.**

Thank you for the suggestion. Although we agree that CUSUM methods are useful for outbreak detection, the current national guidelines indicate the General Department of Preventive Medicine should use the mean plus two standard deviations for the definition of outbreaks. We note this threshold does a good job at detecting outbreaks.

- **Materials and methods should also say where the shapefiles originate.**

The shapefile was obtained through the General Department of Preventive Medicine, Hanoi. We have specified that in the manuscript (page 6, lines 200-20s1).

*“The administrative boundaries of each province were defined using a shapefile provided by the sVietnamese General Department of Preventive Medicine, Hanoi”*

- **Ref 59 for the Worldpop project contains a wrong URL.**

We have amended the URL to:

*“WorldPop. The spatial distribution of population in Vietnam; 2018.* [*https://www.worldpop.org*](https://www.worldpop.org)*”*

- **Why are two different population datasets [refs 59 and 60] used for P in the model and to weight monthly averages of climate variables, respectively?**

The SEDAC population data although gridded, has identical values at each pixel falling within a given administrative district (i.e. admin 2 level). Consequently, it could not be used for the computation of population weighted averages. As a compromise, we used WorldPop estimates for which population estimates vary per pixel.

- **p. 5, l. 161: land-cover variables are per province _and per year_ (please clarify this). I thought they were time-constant until I read they vary annually later in the text.**

We have specified the temporal resolution of the data as follows (page 6, lines 173-179):

*“Total population per province and per year were retrieved from the Socioeconomic Data and Applications Center (SEDAC) Gridded Population of the World project version 4.11 [60] at a 1 km^2^ resolution, 5-yearly for the period 2000 to 2020. Intervening years were generated using linear interpolation. The percentage of urban, peri-urban and rural land-cover per province and per year for the period 2002-2019 was derived from the ESA CCI Land-cover project [56, 61] which describes the land surface into 22 classes at a spatial resolution of 0.00277 degrees.”*

- **p. 5, l. 175: There seem to be two data sources here; where did the data eventually come from? Furthermore, the training period ranged from 2002 to 2006, the testing period from 2007 to 2016, and the forecasting period from April 2020 to September 2020. How does hindcast data for only 2012-2016 fit in here?**

Hindcast data are from the GloSea5 system but were retrieved using the Copernicus Climate Data Store. The mention of 2012 was a typo and has been amended (page 8, lines 239-243).

*“Hindcast data (i.e. historical forecasts) from the GloSea5 system [62, 63] were retrieved using the Copernicus Climate Data Store [56] at monthly time steps for each of the 28 ensemble members for lead times of one to six months ahead, and for the period January 2007 to December 2016. At the time of the computations, data for the period May to October 2016 were unavailable from the Copernicus Climate Data Store.”*

- **p. 6, l. 182: I'd prefer to see "month t = 1, ..., T" instead of the more general "time t" to clarify the temporal resolution of the model.**

We have amended the temporal resolution of time as suggested (page 7, lines 245-24	).

*“Let Y_i,t_ be the number of dengue cases for province i= 1,···,I at month t= 1,···,T where I is the total number of provinces in the data set, and T the total number of time steps for which the model will be run, be modelled using Bayesian generalised linear mixed models (GLMM).”*

- **On Eq (1): Please specify the distribution assumed for the temporal random effects, probably Gaussian.**

We have specified the distribution of the random effects as follows (page 8, lines 176-280):

*“Flat priors were set to regression coefficients (alpha, rho, beta) and penalising complexity priors were assumed for the precision for all random effects [71]. Priors were specified using a Gaussian distribution. We did not explicitly specify a prior distribution for the dispersion parameter (phi). We used the default pc.gamma specification in the INLA R package [72]which assumes a Gamma distribution with values of phi within the interval 1-5668.2.”*

- **p. 6, l. 188: This is a log-linear model with log-transformed past counts, so \rho is not an autoregressive *coefficient* in the usual sense: there is a multiplicative effect (Y_{i,t-1}/P_{i,t-1})^\rho on the mean. Maybe the more general term "autoregressive parameter" should be used for \rho.**

We have amended the specification of ρ as suggested.

- **p. 6, l. 215: I don't understand why annual variation is a reason for the land-cover variables to be excluded from the selection procedure.**

At the population level, the distribution of such factors is unlikely to change at monthly time steps. We wanted to explore the predictive ability of variables that could be associated with monthly fluctuations in dengue cases. We clarify this in the manuscript as follows (page 9, lines 285-287):

*“The best subset of seasonal climate predictors leading to the lowest observed-prediction discrepancies for a given model was obtained using an expanding window time series cross-validation (TSCV) algorithm [53]. Land-cover variables were included in all competing models as they varied annually and are unlikely to change significantly at monthly time steps.”*

- **p. 6, l. 225: n is undefined.**

We have defined *n* as follows (page 9, lines 314-316):

*“Each time step (k), a further month of data was added to the training set until the training set contained n-6 observations where n is the total number of observations in the set.”*

- **p. 7, l. 230: "the best two performing models for each verification metric" doesn't seem correct. The results show only 5 models, so it seems only the top model for each verification metric has been selected. But then in Table 1, model 2 seems to be best for both RMSE and MAE, so how obtain five component models? Please clarify the selection procedure.**

In the previous version of the manuscript, multiple models were within the top-2 performing models for more than one verification metric. Consequently, we ended up with five models. In this revised version, we also have five competing models although this time they correspond to all models with a CRPS value below 90.

- **15. Eq (4) and (5): It seems there is some confusion with indices i and k. For example, M_k in (4) should be M_i. Furthermore, why is w_i a vector and how does it sum to 1 given (5)? The "ML or DIC" part isn't quite clear.**

We removed equations 4 and 5 in the previous manuscript as we are no longer using Bayesian moving averaging for the generation of the superensemble (page 11, lines 362-366).

*“Given a number of competing models, we generated a model superensemble [37] stacking models according to their CRPS. Stacking involves averaging point estimates from multiple models using weighted averages. We found model stacking outperformed Bayesian moving average. Therefore, this technique was adopted for the final version of the system.”*

- **Eq (6): CRPS_f and CRPS_b are undefined.**

We have defined CRPS_f_ and CRPS_b_

- **17. p. 9, l. 314-330: This description is repetitive and should be dropped. The results section should concentrate on results, not methods.**

We have dropped lines 314 to 330 as suggested.

- **18. Figure 7: Shouldn't this be based on the 95% instead of the 75% quantile since the former had better scores?**

We have based the new figure on the best performing outbreak threshold.
