# Supplementary material for: Probabilistic seasonal dengue forecasting in Vietnam: A modelling study using superensembles
Source: PLoS Med. 2021 Mar 4;18(3):e1003542. doi: 10.1371/journal.pmed.1003542 (PMC7971894; doi:10.1371/journal.pmed.1003542)
Supplement: Attachment — Submitted filename: Response_to_reviewers_rev2.docx [file pmed.1003542.s009.docx]

**Response to editor and reviewer comments**

**Manuscript reference number:** PMEDICINE-D-20-02989R1

**Title:** Probabilistic seasonal dengue forecasting in Vietnam: A modelling study using superensembles

We wish to thank the editors and the reviewers for their valuable comments on our manuscript which have led to several improvements to the analysis and manuscript. All changes are described below and can be summarised as follows:

- We incorporated quantitative data on the predictive ability of the model superensemble and the competing models in the Abstract and the Results.
- The notation corresponding to the specification of the model superensemble has been corrected.
- We clarified the use of a conditional Negative Binomial distribution.
- Digits were added to Table 1 to make between-model differences more evident.
- We replaced the term “ROC curve analysis” with “signal detection analysis”.

**Requests from the editors:**

1. **Please address the reviewer’s additional comments below on the description of your methodology.**

We have addressed the additional comments made by the reviewers. We specify the changes to the manuscript below.

2. **Please include line numbers in the margin throughout your text, including the abstract and author summary.**

Number lines have been added throughout the text including the abstract and author summary.

3. **Abstract: Please include some summary quantitative data on the fit of your model to prior data and on your future predictions (with 95% CIs and p values, as appropriate).**

We added quantitative data on the fit of the model superensemble based on historical data and future predictions to the Abstract (pages 1-2, lines 45-57).

*“Using historical forecasts, the superensemble made slightly more accurate predictions (continuous rank probability score, CRPS = 66.8, 95% CI 60.6—148.0) than a baseline model which forecasts the same incidence rate every month (CRPS = 79.4, 95% CI 78.5—80.5) at lead times of one to three months albeit with larger uncertainty. The outbreak detection capability of the superensemble was considerably larger (69%) than that of the baseline model (54.5%). Predictions were most accurate in southern Vietnam, an area that experiences semi-regular seasonal dengue transmission. The system also demonstrated added value across multiple areas compared to previous practice of not using a forecast. We use the system to make a prospective prediction for dengue incidence in Vietnam for the period May to October 2020. Prospective predictions made with the superensemble were slightly more accurate (CRPS = 110, 95% CI 102—575) than those made with the baseline model (CRPS = 125, 95% CI 120—168) but had larger uncertainty.”*

Additionally, we added the following text to the Results section (page 15, lines 620-623)

*“The posterior mean of the predictions made with the superensemble was slightly more accurate (CRPS = 110, 95% CI 102-575) than the posterior mean of the predictions made with the baseline model (CRPS = 125, 95% CI 120-168) albeit with larger uncertainty.”*

**Comments from the reviewers:**

**Reviewer 2:**

- **Thank you for addressing my previous comments. It's a nice article, well done**

We thank the reviewer for the comments on the previous version and this positive evaluation of our manuscript.

**Reviewer 4:** 

- **The authors have revised their paper and this now represents a substantially improved manuscript, acceptable for publication**

Thank you for the positive comments.

**Reviewer 5:**

- **My main concern remains how k-step ahead forecasts are generated. Since forecasting is the main purpose of this manuscript, I think the corresponding description should be improved. I admit that I didn't have the time to look at the revised version in every detail. Nevertheless, I stumbled over a few issues in the authors' reply, which I would like to bring up for consideration.**

We thank you for the opportunity of clarifying the information in the manuscript. We provide an answer to your questions below.

- **Eq 3+4: The math notation is too sloppy for a Bayesian inference approach. I've preferred the previous simplistic textual description of assuming a NegBin for Y_it. Now a random variable $Y$ (without $_{i,t}$) is introduced, whose meaning is undefined, and appending y >= 0 doesn't make any sense. This Bayesian model should be either formulated with mathematical rigour, or with just the core part, i.e., Eq. 4, i.e., the conditional mean of $Y_{i,t}$.**

We have removed Equation 3 from the manuscript, and modified the notation of the models as in the previous submission as suggested (page 8, lines 338-356)

*“Let Y_i,t_ be the number of dengue cases for province i = 1, … , I and time t = 1, … , T where I is the total number of provinces in the data set, and T is the total number of time steps for which the model is fitted using Bayesian generalised linear mixed models (GLMM). Models were fitted with a conditional negative binomial distribution given the number of dengue cases in the previous time step. The general algebraic definition of the models at the linear predictor scale is given by:*

*where α corresponds to the intercept; log(Pi,a[t]) denotes the logarithm of the population at risk for province i and year a[t], included as an offset to adjust case counts by population; log(Yi,t-1+1) denotes the logarithm of the observed number of dengue cases in the previous month plus one with an autoregressive parameter ρ; X is a matrix of k seasonal meteorological explanatory variables with regression coefficients β; L is a matrix of j land-cover (periurban and urban) variables with regression coefficients Є. Long-term trends are modelled using province-specific unstructured random effects for each year (γi,a[t]). Seasonality is accounted for by using province-specific structured random effects for each calendar month (δi,m[t]) with first order auto-regressive prior to allow each month to depend on the previous one.* *Unknown confounding factors, such as interventions and spatial dependency structures representing, for example, human mobility, were incorporated using structured (νi) and unstructured (ui) random effects for each province i.*

- **Eq. 3 would imply that this paper assumes a marginal NegBin for $Y_{i,t}$, but my previous reading suggests that a conditional NegBin was assumed, in particular also conditioning on the past observations just like for other covariates. Please clarify.**

We have clarified that the assumption of a conditional Negative Binomial distribution. (page 8, lines 338-342)

*“Let Y_i,t_ be the number of dengue cases for province i = 1, … , I and time t = 1, … , T where I is the total number of provinces in the data set, and T is the total number of time steps for which the model is fitted using Bayesian generalised linear mixed models (GLMM). Models were fitted with a conditional negative binomial distribution given the number of dengue cases in the previous time step.”*

- **3. Eq. 4 now says that $\rho$ works on past counts not past incidence. However, the text below still contains Y/P. Please check.**

Thank you for highlighting this. We have amended the text as follows (page 8, lines 346-347):

*“log(Yi,t-1+1) denotes the logarithm of the observed number of dengue cases in the previous month plus one with an autoregressive parameter ρ”*

- **The reply confirms that k-step-ahead (k>1) forecasts as implemented here do not carry forward the uncertainty of one-step-ahead forecasts. My interpretation is that the authors eventually assume a NegBin distribution for their forecast $Y_{t+2} | Y_t, ...$, by *imputing* a point prediction for $Y_{t+1}$ to actually compute $Y_{t+2} | Y_{t+1}, ...$. AFAIK, $Y_{t+2} | Y_t$ follows a *mixture* of NegBin distributions (which is not necessarily NegBin), see, e.g.,** [**https://doi.org/10.1016/j.ijforecast.2020.07.002**](https://doi.org/10.1016/j.ijforecast.2020.07.002) **(Section 3.4) for a correct approach. I understand that it is too late to fix this, but it would still be possible to include a cautious comment that all forecasts (including for k > 1) assume a conditional NegBin distribution, imputing one-step-ahead point predictions for lagged counts. I don't know if there is a name for such "naive" forecasts.**

Thank you for highlighting this important point and for the very useful reference. We have added the following comment to the “Prospective lagged dengue cases” subsection (page 10, lines 438-444):

*“We then predicted the number of dengue cases for time t+1. The logarithm of the posterior mean of the predicted number of cases at time t+1 was then used as log(Y_i,t-1_) for time t+2. We repeated these steps for each lead time in the forecast until time t+6. Note we assumed a conditional negative binomial distribution, to generate one-step-ahead point predictions for the reconstructed lagged dengue time series. Once the reconstructed lagged dengue time series was complete, we fitted all competing models as indicated in the Model Selection Section”.*

Additionally, we have added the following sentences to the Discussion (page 19, lines 758-761):

“*Finally, our forecasts do not carry forward the uncertainty of the one-step-ahead forecasts used to simulate prospective lagged cases. Future developments may represent this uncertainty more accurately by*

*using simulation-based path forecasts in a Bayesian framework [91].”*

- **Table 1 should use two digits in the Bias column.**

We have now used two digits for bias and sharpness in Table 1 (page 14):

- **A "ROC curve" analysis would be used to choose a diagnostic threshold and display its effect on TPR and FPR and to display the overall diagnostic accuracy as the area under the ROC curve. I'm thus puzzled by the use of the term "ROC curve" analysis in the manuscript**.

Thank you for this observation. We have replaced “ROC curve analysis” with “Signal detection analysis” which is better suited (page 15, lines 603).
