# Supplementary material for: Probabilistic seasonal dengue forecasting in Vietnam: A modelling study using superensembles
Source: PLoS Med. 2021 Mar 4;18(3):e1003542. doi: 10.1371/journal.pmed.1003542 (PMC7971894; doi:10.1371/journal.pmed.1003542)
Supplement: Attachment — Submitted filename: Response_to_editorial_requests.docx [file pmed.1003542.s010.docx]

**Response to editor and reviewer comments**

**Manuscript reference number:** PMEDICINE-D-20-02989R1

**Title:** Probabilistic seasonal dengue forecasting in Vietnam: A modelling study using superensembles

We wish to thank you for your comments as they have led to improvements to the manuscript. All changes are described below and can be summarised as follows:

- Updated the contact information for others to gain access to the dengue data.
- Ensured that the quantitative results mentioned in the Abstract are also presented in the Results section, and that their values match.
- Briefly discuss dengue vaccination in the Introduction.
- Mention that our study did not require a separate ethical approval in the Methods section.
- Amended line 749 as requested.
- Provide a URL for reference 6.
- Use a different appropriate source for reference 91.

**Requests from the editors:**

1. **Data Availability Statement (DAS): Note that a study author cannot be the contact person for a dataset. Please provide alternative details for your dengue data.**

We have updated the contact information for others to gain access to the dengue data set. The new contact details are as follows:

*Dr Pham Hung, Head of Disease Control Department, Vietnam. Email:* [*hungvncdc@gmail.com*](mailto:hungvncdc@gmail.com)

2. **Abstract: Please ensure that all quantitative data are also presented in the main Results section, and that the values match.**

We have ensured that the quantitative results mentioned in the Abstract are presented in the Results section, and that these values match. Values can be found in page 16, lines 632-639.

*For the historical period (i.e. August 2002 to April 2020), the superensemble made slightly more accurate predictions (continuous rank probability score, CRPS = 66.8, 95% CI 60.6-148.0) than a baseline model which forecasts the same incidence rate every month (CRPS = 79.4, 95% CI 78.5-80.5) at lead times of one to three months albeit with larger uncertainty. For the period May to October 2020, the posterior median of the predictions made with the superensemble was also slightly more accurate (CRPS = 110, 95% CI 102-575) than the posterior median of the predictions made with the baseline model (CRPS = 125, 95% CI 120-168) albeit with larger uncertainty.*

3. **Throughout, please perhaps replace “skill” with a more appropriate term (e.g. ‘strength’), when describing the performance of the forecast system.**

We have replaced the word “skill” with “predictive ability” to describe the performance of the system (lines 33, 238, 338, 482, 484, 591, 594, 599, 602-605, 607, 609, 612-613, 622, 713, 717, 731, 741, 801).

4. **In the introduction, please mention dengue vaccination - although a complex and not very satisfactory situation, this could be discussed.**

We briefly discuss vaccination in the Introduction as follows (Page 3, lines 106-112):

*A live, attenuated, tetravalent dengue vaccine has demonstrated efficacy in two large-scale phase III trials [7] and has now been licensed. However, while the vaccine shows high levels of protection against disease in people with previous exposure to dengue viruses, it may increase the risk of severe dengue if given to seronegative individuals [7]. A lack of reliable and scalable tests for seropositivity currently hinders wider rollout of the Dengvaxia® vaccine and so most countries still primarily rely on insecticide-based mosquito control interventions to limit dengue virus transmission [8].*

5. **Please mention in your Methods that your study did not require separate ethical approval.**

The following text has been included to the Methods section (page 6, line 256):

*Our study did not require a separate ethical approval.*

6. **Line 749: "explicitly incorporate".**

Line 749 has been amended as requested.

7. **Please provide a URL or DOI for reference 6.**

The corresponding URL has been added to Reference 6

*World Health Organization. Background Paper on dengue vaccines. Geneva, Switzerland: World Health Organization; 2018. Available from: https://www.who.int/immunization/sage/meetings/2018/april/2_DengueBackgrPaper_SAGE_Apr2018.pdf*

8. **RE reference 91 listed as “in press”, papers cannot be listed in the reference list until they have been accepted for publication or are otherwise publicly accessible (for example, in a preprint archive). The information may be cited in the text as a personal communication with the author if the author provides written permission to be named. Alternatively, please provide a different appropriate reference.**

Reference 91 has been replaced by a pre-print of the manuscript available through arxiv and is now Reference 92 in the manuscript.

*Bracher J, Held L Endemic-epidemic models with discrete-time serial interval distributions for infectious disease prediction. arXiv:1901.03090v3 [Preprint]. 2019. [posted 2019 Jan 10; revised 2019 Oct 4; revised 2020 Mar 13]. Available from: https://arxiv.org/abs/1901.03090.*

**Comments from Reviewers:**

**Reviewer #5: Thank you for addressing my previous comments, especially for clarifying k-step-ahead as imputed one-step-ahead forecasts and including cautious comments about that. In my opinion, this manuscript is now ready for publication. Congratulations!**

We thank you again for your input as it helped us improve our manuscript.
